# Identification of QR Code Perspective Distortion Based on Edge Directions and Edge Projections Analysis

**DOI:** 10.3390/jimaging6070067

**Published:** 2020-07-10

**Authors:** Ladislav Karrach, Elena Pivarčiová, Pavol Božek

**Affiliations:** 1 Technical University in Zvolen, Faculty of Technology, Department of Manufacturing and Automation Technology, Masarykova 24, 960 01 Zvolen, Slovakia; karrach@zoznam.sk; 2 Slovak University of Technology in Bratislava, Faculty of Materials Science and Technology in Trnava, Institute of Production Technologies, 917 24 Trnava, Slovakia; pavol.bozek@stuba.sk

**Keywords:** QR Code recognition, Quick Response Code localization, adaptive thresholding, finder pattern, perspective distortion, edge projections

## Abstract

QR (quick response) Codes are one of the most popular types of two-dimensional (2D) matrix codes currently used in a wide variety of fields. Two-dimensional matrix codes, compared to 1D bar codes, can encode significantly more data in the same area. We have compared algorithms capable of localizing multiple QR Codes in an image using typical finder patterns, which are present in three corners of a QR Code. Finally, we present a novel approach to identify perspective distortion by analyzing the direction of horizontal and vertical edges and by maximizing the standard deviation of horizontal and vertical projections of these edges. This algorithm is computationally efficient, works well for low-resolution images, and is also suited to real-time processing.

## 1. Introduction

Two-dimensional (2D) matrix codes are a way how to efficiently store data that are machine readable. Thanks to the great spread of smartphones, 2D matrix codes have found application in many areas of life and industry. QR (quick response) Code was invented in 1994 by Denso Wave for the automotive industry in Japan, but nowadays has much wider usage. They are widely used in segments such as manufacturing, logistics, sales, media, advertising, tourism, e-commerce, identification, and authentication [1,2]. The QR Code often contains additional information about the product, the object or the place where it is located. However, they can also be a URL to a web page, Global Positioning System (GPS) coordinates, contact details, delivery address, payment instructions, etc.

**Figure 1:**
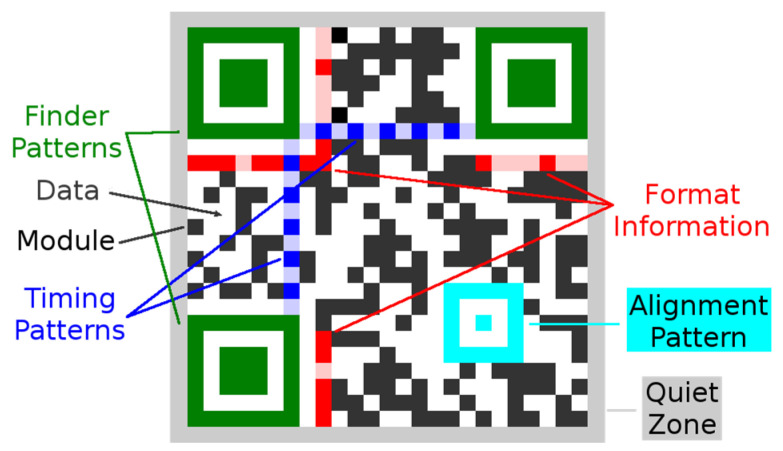
Version 2: 25 × 25 modules Quick Response (QR) Code.

QR codes belong to a group of 2D matrix codes (similarly the data matrix codes). Traditional QR code (QR code Model 1 and Model 2) has a square shape and on its three corners are typical square-shaped patterns—finder patterns (FP), which are used to locate the code and to determine its dimensions and rotation. QR Code is structured as follows (Figure 1).

- Module—the smallest building element of a QR Code represented by a small dark or light square. One module represents one bit: usually, dark module for the “1” and light module for the “0”. Modules are organized into rows and columns and form squared matrix.
- Finder patterns—also called position detection patterns, these are localized in three corners of a QR Code. Each Finder Pattern is formed by an inner dark square (of size 3 × 3 modules) surrounded by a dark frame (of size 7 × 7 modules). Finder Patterns are separated from the rest of a QR Code by a light area of width one module. Finder Patterns are used by readers to determine position and orientation of a QR Code.
- Timing patterns—these are placed inside a QR Code and interconnect finder patterns. Timing patterns are formed by sequence of alternating dark and light modules. The timing pattern is used to determine the size of a module, the number of rows and columns, and possible distortion of a code.
- Alignment patterns—there may be none or more alignment patterns according to a version of a QR Code (QR Code version 1 has no alignment pattern). They allow the scanning device to determine the possible perspective distortion of the QR Code image.
- Format information—this contains additional information such as used error correction level (4 options) or a mask patterns number (7 options), which are required for decoding a QR Code.
- Quiet zone—this is a white area of width at least four modules located around a QR Code (in practice, the width is often less than four modules as required by the standard). The quiet zone should not contain any patterns or structures which can confuse readers.
- Data—they are encoded inside a QR Code and are protected by an error correction carried out via a Reed–Solomon algorithm (allows restoration of damaged data). This also means that a QR Code can be partially damaged and can still be entirely read out. QR Codes provide four user selectable levels of error correction: L (Low), M (Medium), Q (Quartile), and H (High). It means that up to approximately 7%, 15%, 25%, and 30% of the code words, which are damaged, can be restored [3]. Increasing the level of error correction reduces the available data capacity of a QR Code.

QR Codes are available in 40 different versions (version 1 represents a QR Code with 21 × 21 modules and version 40 represents QR Code with 177 × 177 modules; the relation between size and version can be expressed as: *Size* = 21 + (*Version* − 1) × 4).

Each QR Code version has the maximum data capacity according to the amount of data, character type and error correction level. For example, there is capacity of 10 alphanumeric (alt. 17 numeric) characters in a QR Code of minimal version 1 (size 21 × 21 modules), and capacity up to 1852 alphanumeric (alt. 3057 numeric) characters in maximal version 40 (size 177 × 177 modules) at highest error correction level [3]. QR Codes are able to encode numeric, alphanumeric, Kanji characters and also binary data.

The rest of this paper is organized as follows: in Section 2 a brief overview of previously published methods is presented, in Section 3 two finder pattern localization methods are summarized, in Section 4 the proposed method for QR Code perspective distortion identification is introduced, and in Section 5 the experimental results are presented.

## 2. Related Work

There were various approaches for QR Code detection in images published in the past. We can divide them into three main groups.

### 2.1. Finder Pattern-Based Localization Methods

The first group uses for location of a QR Code features of three typical finder patterns (Figure 1 and Figure 3). 

The shape of the finder pattern (square in the frame, Figure 3) was intentionally selected by QR Code inventors because “it was the pattern least likely to appear on various business forms and the like” [4]. They surveyed innumerable examples of printed matter and came up with the least used ratio of black and white areas on printed matter. This ratio was 1:1:3:1:1 (B:W:BBB:W:B), regardless of an angle of scanning.

In Lin and Fuh [5] a binary image is scanned horizontally and then vertically to find all points matching the ratios 1:1:3:1:1. Then the points belonging to the same FP are combined and the angle between three FP is checked.

In Li et al. [6] the connected components in a binary image are compressed using run-length coding on row basis. Then typical ratios of FP are searched row by row. Found FPs establish minimal containing region in which foreground points are searched for ratios 1:3:1, using a modified Knuth–Morris–Pratt algorithm for fast exploration, to compute a centre coordinate of FP.

In Belussi and Hirata [7] they use a cascade of weak classifiers trained on the finder patterns of the QR Code (The Viola-Jones’ rapid object detection method and Haar-like features extracted under floating window are used). In the second stage, the geometrical relationships among detected candidate finder patterns are analyzed in order to verify if there are subgroups of three of them spatially arranged as three corners of a square region.

In Bodnár and Nyúl [8] they also use cascade classifiers using Haar-like features, local binary patterns and histograms of oriented gradients, trained for the finder patterns of QR Codes and for the whole code region as well (also on multiple scales). The training process was too time-consuming.

In Tribak and Zaz [9] the first horizontal and vertical scans are performed to localize preliminarily QR Code patterns, followed by the principal component analysis (PCA) method, which allows removing false positives.

In Tribak and Zaz [10] instead of PCA, the authors use 7 Hu invariant moments as feature descriptors to preliminarily localized FP. The obtained feature vector is compared with feature vectors of set of training samples using Euclidean distance.

### 2.2. Local Features with Region Growing-Based Localization Methods

The methods in the second group extract specific features from an image under a floating window and these features are checked against sample features. The regions of an image, which are classified as QR Code candidate regions, are merged into larger ones. Local features may be constructed from the Ciążyński and Fabijańska window histogram [11] or from HOG (histogram of oriented gradients), angles of two main gradient directions, a number of edge pixels and an estimation of probability score of a chessboard-like structure Szentandrási et al. [12].

### 2.3. Connected Components-Based Localization Methods

The third group of methods attempts to detect QR Code as a whole. It is usually based on the fact that a QR Code consists of many small black squares which are relatively close to each other [13,14,15]. Usually, morphological erosion and dilation are applied to connect these small squares into one monolithic object and remove small objects. Gaur and Tiwari [13] proposed to use Canny edge detector together with morphological operations. Kong [14] combined Harris corner detection with convex hull algorithm to get the outline of the outer quadrilateral of a QR Code. In Sun et al. [15] researchers introduce algorithm, which aims to find QR Code area by detection of four corners of 2D barcode. They combine Canny edge detection with contours finding algorithm.

### 2.4. Deep Learning Based Localization Methods

Hansen et al. [16] described how to adapt deep learning based object detection algorithm YOLO for the purpose of detecting barcodes. Similarly, Zharkov and Zagaynov [17] introduced new fast and robust deep learning detector of 1D and 2D barcodes based on semantic segmentation approach. Convolutional neural network was utilized also in works [18,19].

## 3. The Finder Pattern Localization Methods

**Figure 2:**
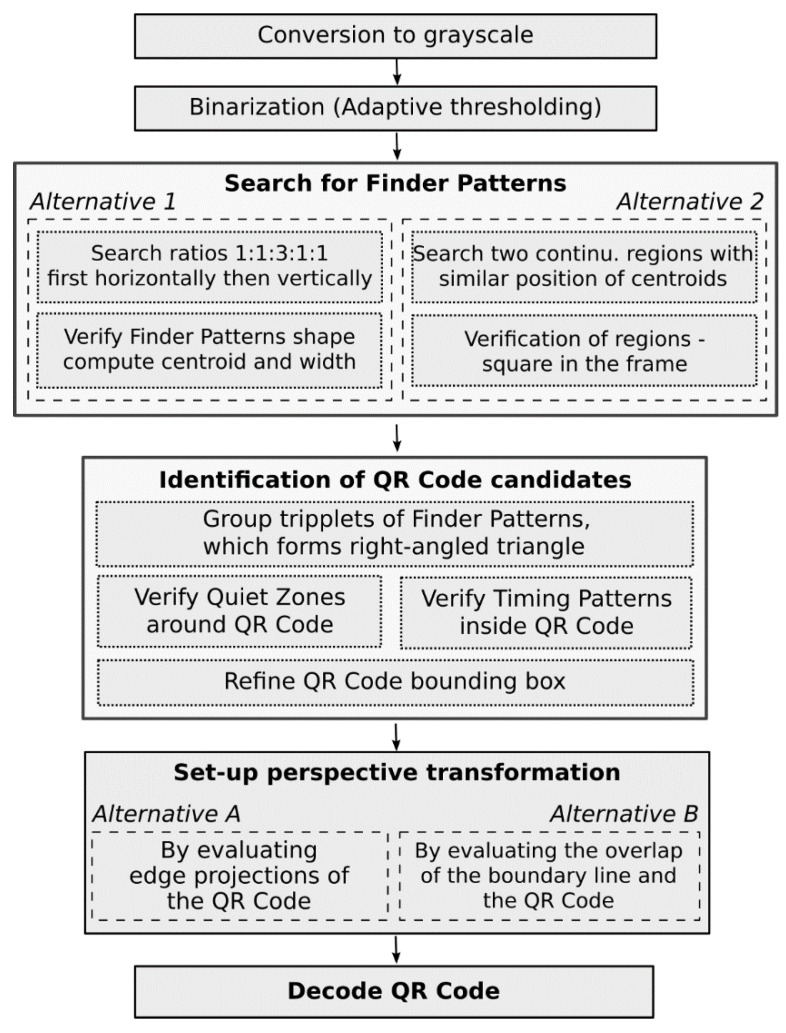
The flow chart of the proposed algorithm.

In the following paragraphs, we will give a brief overview of two methods for locating QR Codes which are based on finding three typical patterns—finder patterns—in the corners of the QR Code [5,20]. These three finder patterns also identify the three corner points of the QR Code bounding box. The method for determining the position of the 4th corner of the bounding rectangle, which we present in Section 4, is the main contribution of this article. The individual steps of the QR Code location algorithm are schematically shown in Figure 2.

Image processing begins with the conversion of the input image to a grayscale image, followed by adaptive thresholding grayscale image to a binary image. The adaptive thresholding techniques [21,22,23,24] is an effective way to separate the dark modules of a QR Code from the light ones, even for images with low contrast or uneven illumination (if classical methods of adaptive thresholding are not sufficient, learning based binarization utilizing convolutional neural networks can be considered [25]).

### 3.1. Finder Pattern Localization Based on 1:1:3:1:1 Search

This method (Alternative 1 in Figure 2) utilizes characteristic feature of Finder Patterns, namely that with any rotation of a QR Code in an image it is possible to put lines through the centre of the finder pattern, so that the dark and light points lying on them will alternate in the ratio 1:1:3:1:1. 

**Figure 3:**
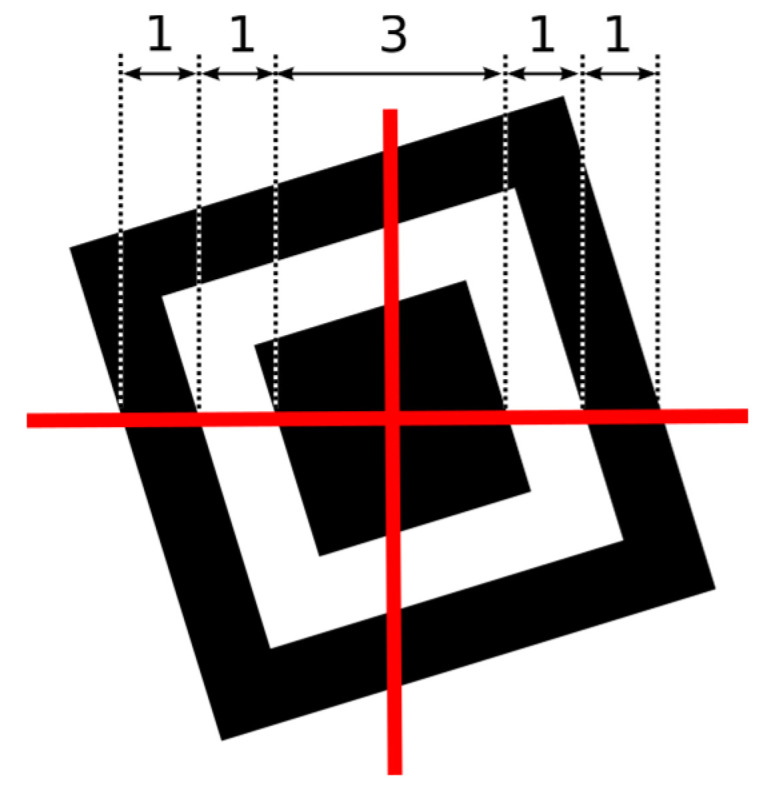
The finder pattern.

This structure has the property that also when it is rotated, it is possible to make such a horizontal and vertical cut that dark and light points will alternate in the ratio 1:1:3:1:1 (Figure 3).

We look for centres of finder patterns by scanning the binary image horizontally, line by line from top to bottom, to find consecutive sequences of black and white points in a line matching the ratios 1:1:3:1:1 (*B*_1_:*W*_2_:*BBB*_3_:*W*_4_:*B*_5_ where *B*_x_, *W*_x_ denotes the number of black, white points). In real images the ratio of black and white points is not ideal, so we have to consider tolerances:

(1) $$\begin{array}{l} B_{1},W_{2},W_{4},B_{5}\in \langle w-1.5,w+1.5\rangle \\ BBB_{3}\in \langle 3w-2,3w+2\rangle \\ BBB_{3}>\max\left(B_{1}+W_{2},W_{4}+B_{5}\right) \end{array},\;\mathrm{where}\;w=\frac{B_{1}+W_{2}+BBB_{3}+W_{4}+B_{5}}{7}$$

**Figure 4:**
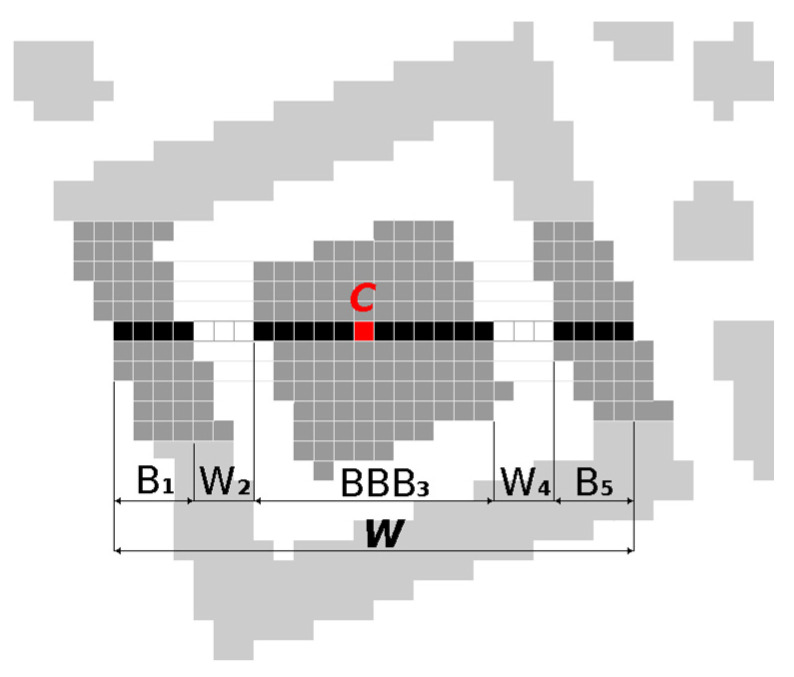
Finder pattern candidate matching 1:1:3:1:1.

For each match we store coordinates of Centroid (*C*) and Width (*W* = *B*_1_ + *W*_2_ + *BBB*_3_ + *W*_4_ + *B*_5_) of the sequence in a list of finder pattern candidates (Figure 4).

Subsequently, we search through this list of candidates and group together candidates whose centroids are at most 3 points horizontally and at most 3/7*W* vertically away from each other. A new centroid *C* and width *W* of the group is set as average of *x*, *y* coordinates of centroids *C* and widths *W* of nearby candidates.

**Figure 5:**
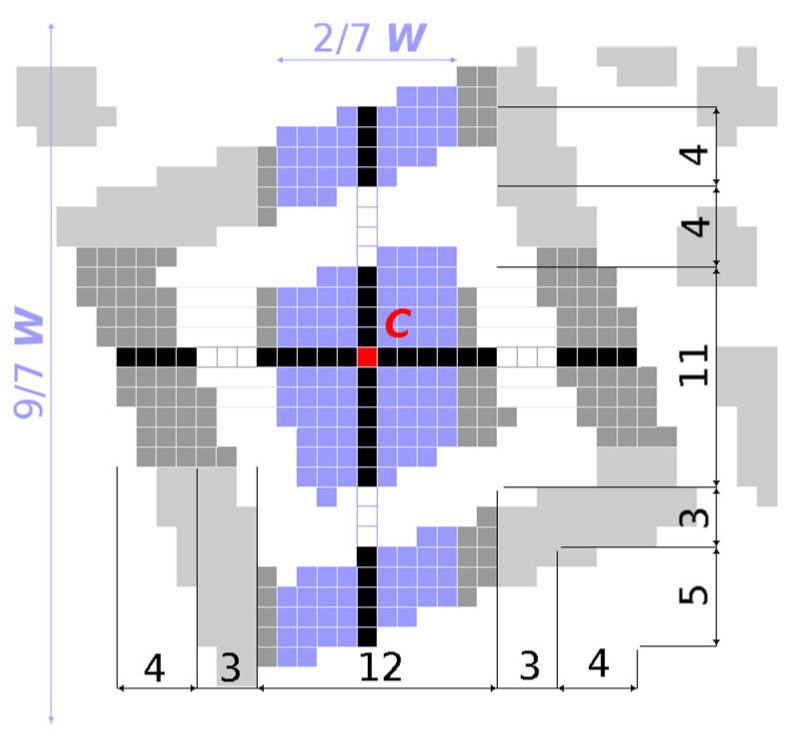
Finder pattern candidate matching 1:1:3:1:1 in both axes.

After the grouping, we verify if also in vertical direction the ratio of black and white points is 1:1:3:1:1 (Figure 5). We do not scan the whole image vertically but only the surroundings of the finder pattern candidates, where the surrounding area is defined as:

(2) $$x\in \langle C_{x}\pm W/7\rangle \;\mathrm{and}\;y\in \langle C_{y}\pm 4.5W/7\rangle$$

Candidates, where there is no vertical match are rejected. 

**Figure 6:**
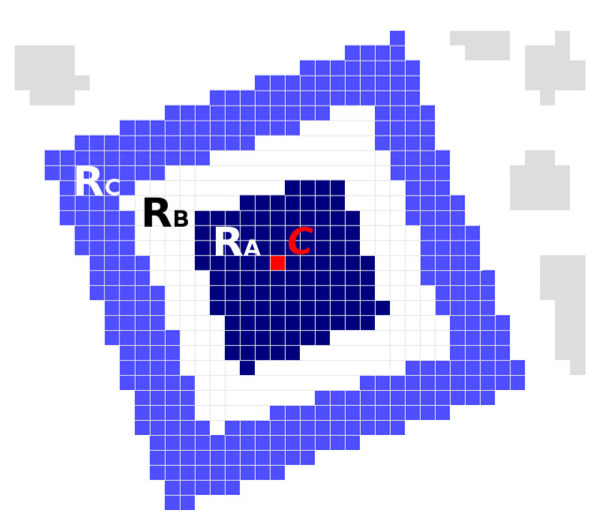
Connected components of the finder pattern candidate.

The finder pattern is built up by 3 × 3 modules square (R_A_), which is placed in the frame of 7 × 7 modules (R_C_) (Figure 6). The individual candidates for the Finder Pattern must therefore also meet all the following conditions:

- Area(R_A_) < Area(R_B_) < Area(R_C_) and Area(R_B_)/Area(R_A_) < 3 and Area(R_C_)/Area(R_A_) < 4
- 0.7 < Width(R_B_)/Height(R_B_) < 1.3 and 0.7 < Width(R_C_)/Height(R_C_) < 1.3
- Distance(Centroid(R_A_), Centroid(R_B_)) < 3 and Distance(Centroid(R_A_), Centroid(R_C_)) < 3

To check the finder pattern candidate and thus obtain the characteristics of the R_A_, R_B_ and R_C_ regions, we apply the flood fill algorithm. We start filling from centroid C (which lies in dark region R_A_) and continue through the light surrounding (region R_B_) to the dark surrounding (region R_C_). During region filling, we compute above mentioned region descriptors:

- Area (*M*_00_)
- Centroid (*M*_10_/*M*_00_, *M*_01_/*M*_00_), where *M*_00_, *M*_10_, *M*_00_ are raw image moments
- Bounding box (Top, Left, Right, Bottom)

The region descriptors are also used to update finder pattern candidate centroid *C* and compute the module width *MW* using equations:

(3) $$C=(\frac{M_{10}(R_{A})+M_{10}(R_{B})+M_{10}(R_{C})}{M_{00}(R_{A})+M_{00}(R_{B})+M_{00}(R_{C})},\frac{M_{01}(R_{A})+M_{01}(R_{B})+M_{01}(R_{C})}{M_{00}(R_{A})+M_{00}(R_{B})+M_{00}(R_{C})})\;MW=\sqrt{M_{00}(R_{A})+M_{00}(R_{B})+M_{00}(R_{C})}/7$$

### 3.2. Finder Pattern Localization Based on the Overlap of the Centroids of Continuous Regions

In this method (Alternative 2 in Figure 2), similar to Alternative 1, three typical position detection patterns, finder patterns, are used to locate a QR Code. However, we utilize another feature of them. The finder pattern consists of a smaller square that is centred in a larger frame. Both of these shapes (dark square and dark frame) form separate continuous components in the image, but they have the same position of their centroids.

To connect adjacent foreground points to continuous regions, a connected component labelling algorithm is applied to the binary image [26,27]. During the run of the algorithm, when a point with *x*, *y* coordinates is added to the region, the descriptor of the continuous region is updated as follows:

- raw moments *M*_00_ ←*M*_00_ + 1, *M*_10_←*M*_10_ + *x*, *M*_01_←*M*_01_ + *y*, which we will use later to calculate the centroid of the area (*C*_x_ = *M*_10_/*M*_00_, *C*_y_ = *M*_01_/*M*_00_);
- bounding box defined by top, left, bottom and right boundary.

(Note: as we do not need an image of the markers but only the continuous region descriptors, we can work with a more efficient modification of the marking algorithm, which works only with a 2-row markers buffer)

In following process, the region descriptors are searched (looking for square areas that could represent either an inner square or an outer frame) and those are selected that meet the conditions:

- Area (*M*_00_) must be at least 9 (minimum inner square size is 3 × 3);
- Aspect ratio width to height of the region must be between 0.7 and 1.3 (here we assume that the QR Code is approximately square sized. In case of a much stretched QR Code this tolerance may be increased).

We determine the centroid of the current region (*C*_x_, *C*_y_) and look for another region with a similar position of centroid (to reduce the number of comparisons, we work with a sorted list of regions by the *x*, *y* coordinates of the centroids or we can use a memory map of the centroids. The memory map is reduced in a 4:1 ratio to original image, which allows small inaccuracies in the position of centroids). There can be a maximum of 2 such continuous regions in the QR Code that could have similar positions of centroids.

If we found a region with a similar position of the centroid, we must verify whether these two regions can represent a smaller square in a larger frame. For region R_C_ representing the outer frame and region R_A_ representing the inner square, the following must apply:

- Area(R_C_) > Area(R_A_) and Area(R_C_)/Area(R_A_) ≤ 4
- Bounding box of region R_A_ must lie entirely within the bounding box of region R_C_
- Distance(Centroid(R_C_), Centroid(R_A_)) < 3

If we find two such regions R_A_ and R_C_, then we calculate the centroid of the finder pattern candidate and the module width *MW* as:

(4) $$C=(\frac{M_{10}(R_{A})+M_{10}(R_{C})}{M_{00}(R_{A})+M_{00}(R_{C})},\frac{M_{01}(R_{A})+M_{01}(R_{C})}{M_{00}(R_{A})+M_{00}(R_{C})})\;MW=\sqrt{\frac{M_{00}(R_{A})+M_{00}(R_{C})}{33}}$$

(The area of region R_A_ is 9 *MW* (3 × 3 *MW*) and the area of region R_C_ is 24 *MW*)

### 3.3. Grouping of Finder Patterns

We have identified candidates for the finder patterns, which are defined by their centroids. Now we must find such triplets from the list of Finder Pattern candidates, which could be the three corners of a QR Code. We build a matrix of distances between centroids and examine all three element combinations of all finder pattern candidates. For each combination, we check if the right-angled triangle can be constructed, so that these conditions are met:

- size of each side must be in predefined interval (the smallest and the largest expected QR Code);
- difference in sizes of two legs must be in tolerance ±14 (for non-distorted, non-stretched QR Code is sufficient less tolerance);
- difference in size of the real and theoretical hypotenuse must be in tolerance ±12 (for non-distorted, non-stretched QR Code is sufficient less tolerance).

**Figure 7:**
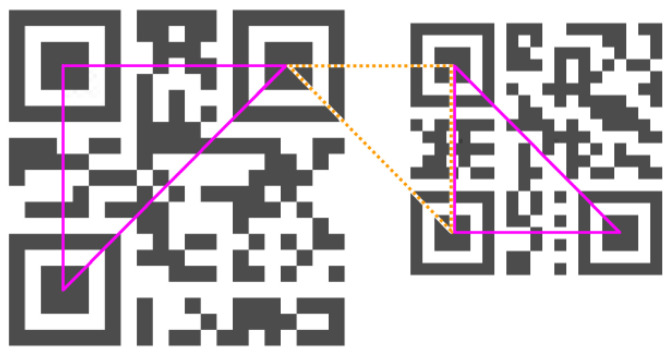
QR Code candidates.

In this way, we have built a list of QR Code candidates (defined by triplet FP_1_, FP_2_, FP_3_) based only on the mutual position of three finder pattern candidates. However, as we can see on Figure 7 (if the image contains multiple QR Codes), not all of the three QR Code candidates are real ones (dotted orange is false positive).

To exclude false positive candidates for a QR Code, defined by a triplet of finder patterns, we verify the presence of the quiet zone around the QR Code and the timing pattern inside the QR Code.

**Figure 8:**
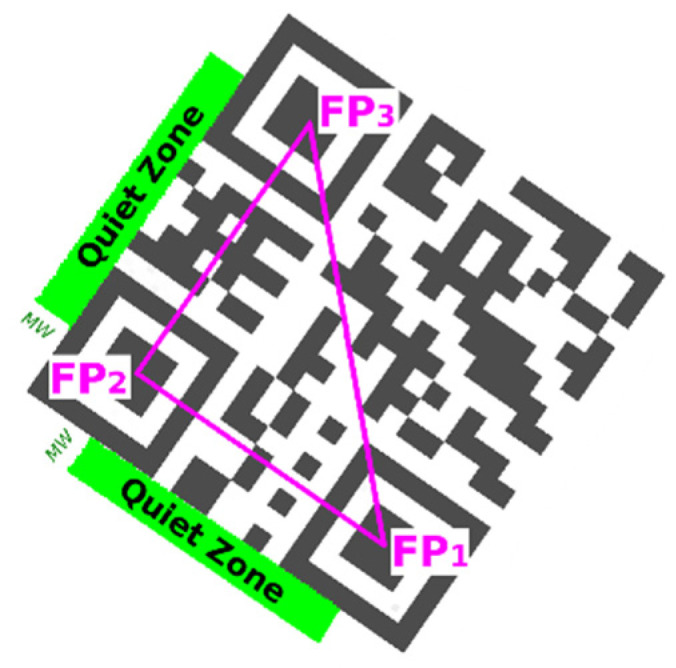
Quiet zones around QR Code.

To verify the quiet zone, we check if there are only white points in the rectangular area of binary image which is parallel to line segments defined by FP_1_–FP_2_ and FP_2_–FP_3_ (Figure 8). For fast inspection of line points we use Bresenham’s line algorithm [28]. 

**Figure 9:**
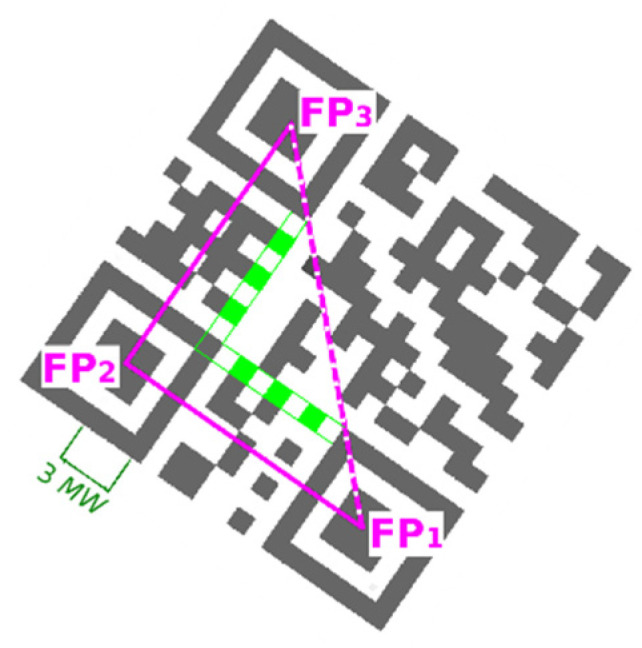
Timing patterns inside a QR Code.

To verify timing patterns inside the QR Code, we examine the rectangular area between inner corners of finder patterns (Figure 9). We count the number of white and black points in the binary image and we calculate average length *L*_AVG_ of white and black line segments and its standard deviation *L*_STDDEV_. The timing pattern is expected to satisfy the following conditions:

(5) $$\left|L_{\mathrm{AVG}}-MW\right|<1\;\mathrm{and}\;L_{\mathrm{STDDEV}}<1$$

### 3.4. QR Code Bounding Box

**Figure 10:**
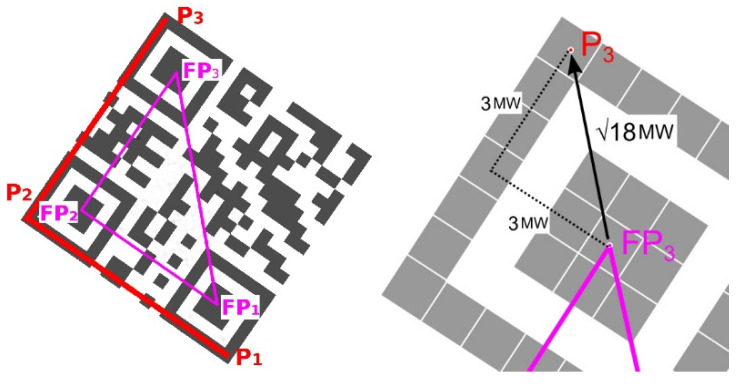
Bounding box.

Vertexes of triangle FP_1_–FP_2_–FP_3_ are centroids of three finder patterns. Then this triangle is extended to the triangle P_1_–P_2_–P_3_, where arms of the triangle go through border modules of the QR Code (Figure 10). For example, the shift of FP_3_ to P_3_ in direction defined by vector (FP_1_, FP_3_) can be expressed as:

(6) $$P_{3}=FP_{3}+\frac{FP_{3}-FP_{1}}{\left|FP_{3},FP_{1}\right|}MW\sqrt{18}$$

where *MW* is module width.

## 4. The Proposed Method

Identification of perspective distortion of the QR Code and determination of the position of the 4th corner point of the QR Code.

In the case of perspective (projective) distorted QR Codes, we need 4 points to set-up perspective transformation from a source square to destination quadrilateral [29]. The 3 corner points P_1_, P_2_, P_3_ have been identified using 3 finder patterns of the QR Code. Now the position of the 4th corner point P_4_ must be determined.

### 4.1. Alternative A—Evaluation of the Edge Projections

**Figure 11:**
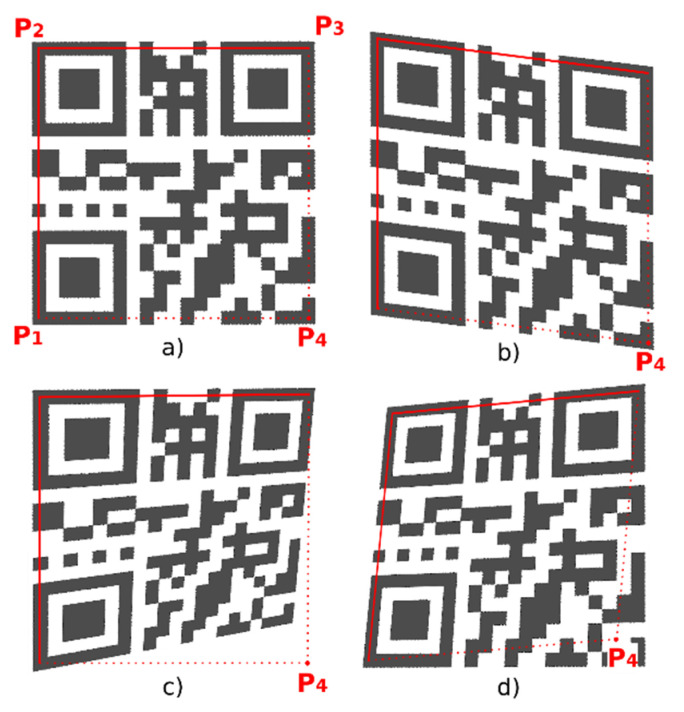
Initial estimate of position of 4th point P_4_. (**a**) undistorted QR Code; (**b**) skewed QR Code; (**c**,**d**) perspective distorted QR Codes.

**Figure 12:**
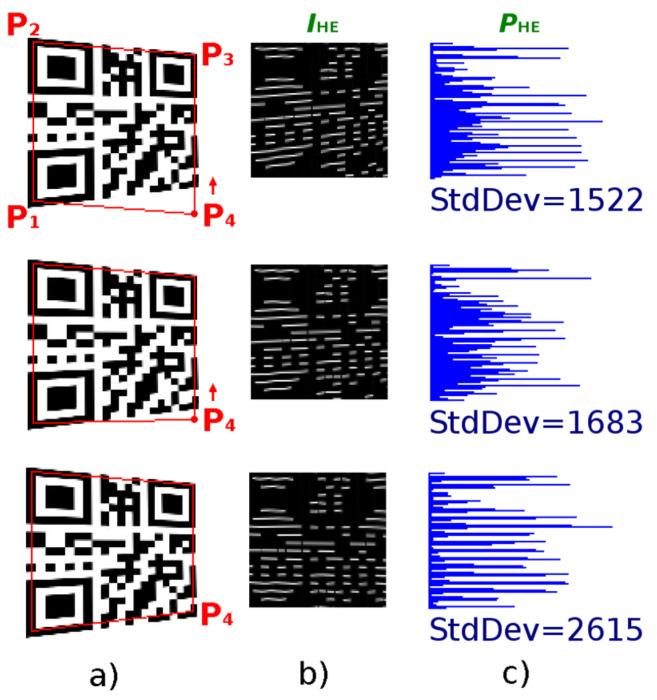
Identification of perspective deformation of QR Code: (**a**) QR Code; (**b**) horizontal edges; (**c**) horizontal projection of horizontal edges.

A QR Code is a 2D matrix and inner structure of the QR Code forms a chessboard-like structure. That means the individual modules create edges that are perpendicular to each other. It can be said that the optimal position of the point P_4_ is such, where the vertical and horizontal edge projections reach the largest standard deviation. We assume that at the optimal position of the point P_4_, both horizontal and vertical edges will be in alignment and thus their projections will show significant local maxima (amplitudes), which will alternate with significantly lower (up to zero) values. In the following, we propose an iterative algorithm to gradually improve the position of point P_4_ in order to find the optimal boundary of the QR Code. Algorithm can be defined by these steps:

Start with an initial estimate of the position of the point P_4_, which is calculated as the intersection of lines parallel to P_2_–P_3_ and P_2_–P_1_, so that points P_1_–P_2_–P_3_–P_4_ form a parallelogram (Figure 11 shows the starting position of the P_4_ point for various distorted QR Codes). Then start moving (shifting) the point P_4_ first vertically in the direction P_4_–P_3_ (Figure 12) and then horizontally in the direction P_4_–P_1_. The initial size of the shift step is for QR Codes with modules greater than 2 points set to 2 points, otherwise it is set to 1 point (the smaller the QR Code module is, the smaller the step is also).

For each shift of point P_4_, from the transformed region *I*_T_ of the grayscale image, defined by points P_1_–P_2_–P_3_–P_4_, determine the image of horizontal edges *I*_HE_ (the horizontal edge is calculated as the difference between the brightness of the current point at coordinates (*x*, *y*) and the point on the previous line at coordinates (*x*, *y*−1): $d_{y}=I_{T}(x,y)-I_{T}(x,y-1)$) and the horizontal projection (sum of absolute values) of these edges in the horizontal direction $P_{\mathrm{HE}}(y)=\sum_{x}\left|d_{y}(x,y)\right|$. For the vector *P*_HE_ calculate the standard deviation (the score).

If the standard deviation (score) increases, then proceed in the selected direction, otherwise if the score has decreased during the last two steps, go back 2 steps (to the position with the highest score) and change the direction of the shift to the opposite. Repeat step 2.

If there is no increase even after the change of direction, return to the position of the point P_4_ with the highest score and reduce the shift step to ½ and repeat steps 3 and 4 (to refine the position of the P_4_ point).

Just as the optimal position of the point P_4_ in the vertical direction was searched using horizontal edges and horizontal projection (Figure 12), in the same way look for its optimal position in the horizontal direction using vertical edges. $d_{x}=I_{T}(x,y)-I_{T}(x-1,y)$ and vertical projection of edges $P_{VE}(x)=\sum_{y}\left|d_{x}(x,y)\right|$.

(Note: to compute edge image, i.e., the derivate of grayscale image, we have tested also other derivate masks like Prewitt operator [−1, 0, +1] and non-local maxima suppression, but experimental results were not significantly better, therefore we stayed on simple difference [−1,+1], which is computationally most effective.)

In Figure 12 we see that by gradually moving the point P_4_ upwards to the point P_3_ (and thus to a more precise delineation of the QR Code), the standard deviation increases in each step and the horizontal projection of the horizontal edges has more pronounced local maxima, which are alternated by more pronounced minima.

**Figure 13:**
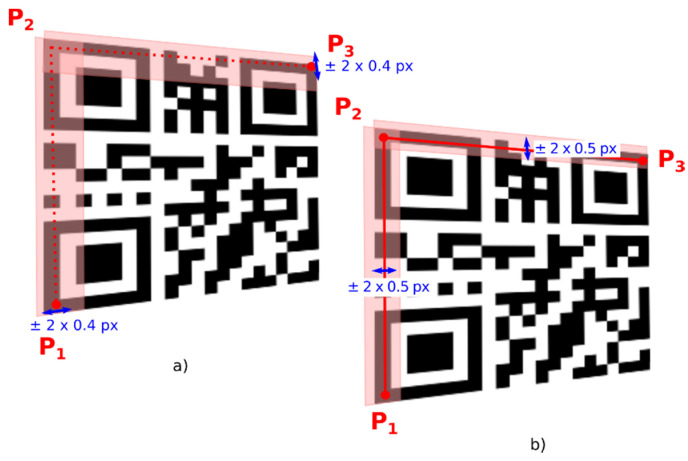
Refining the position of the corner points of the QR Code (alternative 1). (**a**) refining the position of the points P_1_ and P_3_; (**b**) refining the position of the lines P_1_–P_2_ and P_2_–P_3._

As the above mentioned method (Alternative A) is sensitive (especially for small QR Codes with a module size up to 3 points) to the precise alignment of boundary lines P_1_–P_2_ resp. P_2_–P_3_ with the outer edges of the QR Code, it is necessary to refine the positions of these points. This can be achieved using the same approach (by evaluation of edge projections) and by moving the points P_1_ and P_3_ (Figure 13a) and then the lines P_1_–P_2_ and P_2_–P_3_ (Figure 13b) separately, while working with the projections of the edges in a narrow region of the image around the lines given by points P_1_–P_2_ and P_2_–P_3_ respectively. 

**Figure 14:**
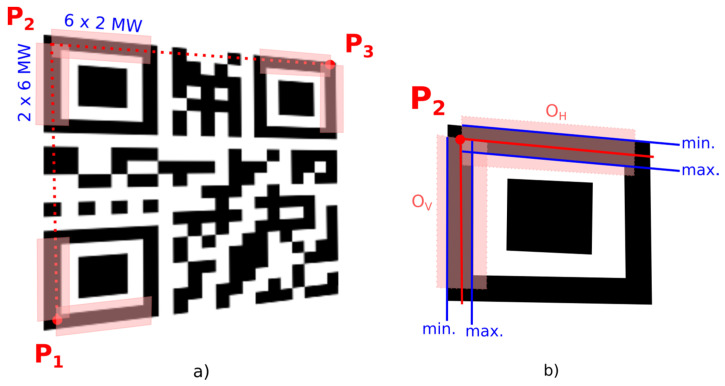
Refining of the position of the corner points of the QR Code (alternative 2). (**a**) areas of the finder pattern where edge projections are evaluated; (**b**) local extrema in edge projections.

Another alternative way to refine the initial position of corner points P_1_, P_2_, P_3_ uses edge projections in the narrow area of the outer frame of the finder pattern and shifts the corner points to the middle between the local minimum (represents white-black transition on the outer edge of the finder pattern frame) and the local maximum (represents black-white transition on the inner edge of the finder pattern frame) of edge projections. Horizontal projections of horizontal edges in the *O*_H_ area are used for centering in the vertical axis and vertical projections of vertical edges in the *O*_V_ area are used for centering in the horizontal axis (Figure 14).

After refining the positions of points P_1_, P_2_, P_3_, we look for the optimal position of point P_4_ as described in Section 4.1.

#### Identification of QR Code Distortion by Edge Orientation Analysis

Before the aforementioned method (Alternative A) it is possible to add one more step in which the orientation (directions) of significant edges in a QR Code is analyzed. According this analysis it is possible to identify perspective or cylindrical distortion of the QR Code and shift the initial position of the 4th point P_4_. (this rough determination of the initial position will allow us to reduce the number of steps required to find the optimal position of point P_4_ in Alternative A as well as to identify the cylindrical distortion. For QR Codes that are cylindrically distorted, Alternative A is not suitable because the perspective transformation is not sufficient to remove this type of deformation).

**Figure 15:**
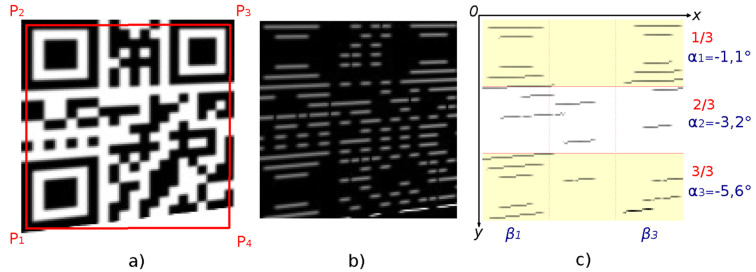
Identification of QR Code distortion by edge orientation analysis: (**a**) QR Code; (**b**) horizontal edges image; (**c**) significant edges.

First, the image of the horizontal edges is analyzed (Figure 15b) and then the image of the vertical edges is analyzed also. The edge images are calculated for the sub-region of the grayscale image, which is defined by points P_1_–P_2_–P_3_–P_4_ (Figure 15a). For edge images the following steps are performed:

Suppress weak edge points in the edge images that have gradient less than the specified threshold (<20) and which do not represent local maxima. In the image of horizontal edges, the point *d*(*x*, *y*) is considered to be a local maximum if: *d*(*x*,*y* − 1) < *d*(*x*,*y*) > *d*(*x*,*y* + 1) and in the image of vertical edges if: *d*(*x* − 1,*y*) < *d*(*x*,*y*) > *d*(*x* + 1,*y*).

Connect adjacent strong edge points into continuous regions. For each contiguous region calculate the region descriptor: raw moments of zero to second order: *M*_00_ (area), *M*_10_, *M*_01_, *M*_20_, *M*_11_, *M*_02_ (Equation (7)).

(7) $$M_{ij}=\sum_{x}\sum_{y}x^{i}y^{j}I_{E}(x,y)$$

where *x*, *y* are coordinates of strong edge point in the edge image *I*_E_.

Filter out insignificant regions (short edges) which have area *M*_00_ less than 3 times the estimated size of the module *MW* (Equations (3) and (4)). Continue to work only with significant regions (edges) (Figure 15c).

Divide the edge image horizontally and vertically into thirds. Classify each region into one of these thirds, based on the location of the centroid of each individual region. Centroid *C* of the region is calculated according to Equation (8). For each third calculate the average angle from the angles of the individual regions, which are classified into the given third. The angle (orientation) *α* of the region is also calculated from the moments (Equation (9)).

(8) $$C=\left(\frac{M_{10}}{M_{00}},\frac{M_{01}}{M_{00}}\right)$$

(9) $$\alpha =\frac{1}{2}\arctan\left(\frac{2\mu_{11}}{\mu_{20}-\mu_{02}}\right)$$

where *μ* are central moments of second order, which can be calculated from raw moments *M*.

Evaluate the average angles of regions in individual thirds.

Divide the image of horizontal edges horizontally into thirds and also the image of vertical edges vertically into thirds. If the average angle in the individual thirds gradually decreases or increases, i.e.,

$\alpha_{1}<\alpha_{2}<\alpha_{3}$ or $\alpha_{1}>\alpha_{2}>\alpha_{3}$, where *α*_1_ is average angle in 1st third, *α*_2_ is average angle in 2nd third and *α*_3_ is average angle in 3rd third.

It is then a perspective-distorted QR Code and the position of point P_4_ can be shifted (either vertically in the case of analysis of horizontal edges or horizontally in the case of analysis of vertical edges) by:

$l\tan(\alpha_{3})$, where *l* represents the width in the case of the image of horizontal edges and the height of the image in the case of the image of vertical edges.

Divide the image of the horizontal edges vertically into thirds and mark the average angle in the left third as *β*_1_ and the average angle in the right third as *β*_3_. If the angles *β*_1_ and *β*_3_ have opposite signs and their difference is above a defined threshold, then this indicates a cylindrical distortion in the *y*-axis. Similarly, we identify the cylindrical distortion in the *x*-axis by dividing the image of the vertical edges horizontally into thirds and comparing the angles in the upper and lower thirds.

### 4.2. Alternative B—Evaluation the Overlap of the Boundary Line

**Figure 16:**
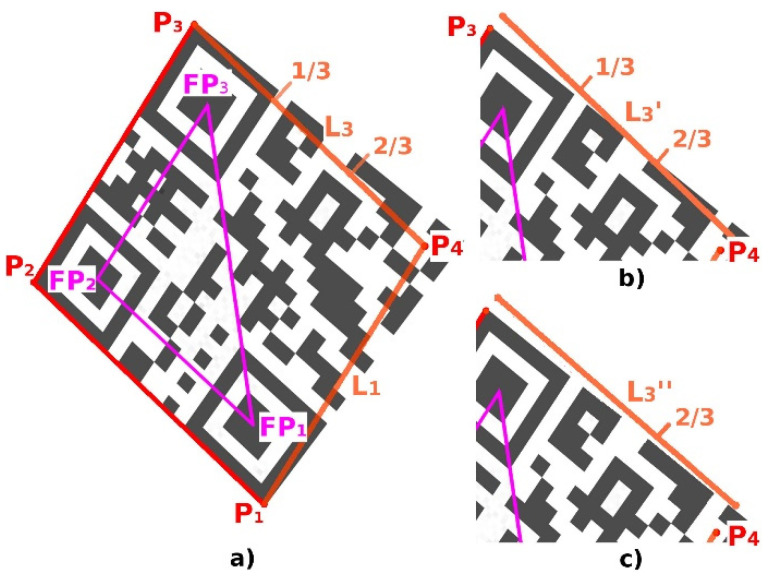
Perspective distortion. (**a**) initial position of the point P_4_ and lines L_1_, L_3_; (**b**) first shift of the line L_3_; (**c**) second shift of the line L_3._

Another method (Alternative B) how to find the optimal position of the 4th corner point P_4_ is based on moving the boundary lines P_3_–P_4_ and P_1_–P_4_ and evaluating the overlap of the boundary lines with a QR Code (Figure 16).

**Figure 17:**
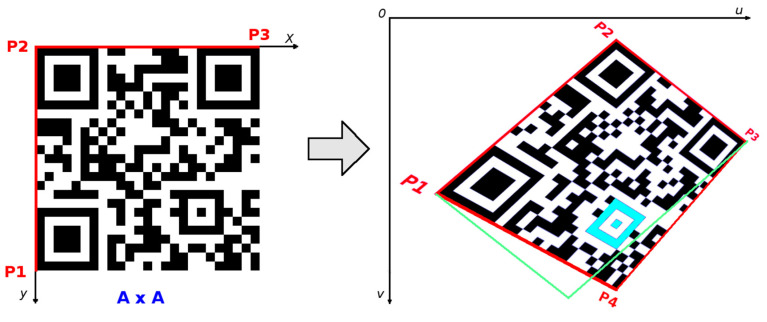
Perspective transformation.

Once we have identified the position of the 4th point, P_4_, we can set-up perspective transformation from the source square domain representing an ideal QR Code to the quadrilateral destination representing a real QR Code in the image (Figure 17).

Using the following equations [29]:

$u=\frac{ax+by+c}{gx+hy+1}$, $v=\frac{dx+ey+f}{gx+hy+1}$, where coefficients can be computed from coordinates of points P_1_(*u*_1_,*v*_1_), *P*_2_(*u*_2_, *v*_2_), *P*_3_(*u*_3_, *v*_3_), *P*_4_(*u*_4_, *v*_4_) as:

(10) $$a=\left(u_{3}-u_{2}\right)/A+gu_{3},\;b=\left(u_{1}-u_{2}\right)/A+hu_{1},\;c=u_{2}\;d=\left(v_{3}-v_{2}\right)/A+gv_{3},\;e=\left(v_{1}-v_{2}\right)/A+hv_{1},\;f=v_{2}\;g=\frac{\left|\begin{array}{cc} du_{3} & du_{2}\\ dv_{3} & dv_{2} \end{array}\right|}{\left|\begin{array}{cc} du_{1} & du_{2}\\ dv_{1} & dv_{2} \end{array}\right|},\;h=\frac{\left|\begin{array}{cc} du_{1} & du_{3}\\ dv_{1} & dv_{3} \end{array}\right|}{\left|\begin{array}{cc} du_{1} & du_{2}\\ dv_{1} & dv_{2} \end{array}\right|}\;du_{1}=(u_{3}-u_{2})A,\;du_{2}=(u_{1}-u_{4})A,\;du_{3}=u_{2}-u_{3}+u_{4}-u_{1}\;dv_{1}=(v_{3}-v_{2})A,\;dv_{2}=(v_{1}-v_{4})A,\;dv_{3}=v_{2}-v_{3}+v_{4}-v_{1}$$

### 4.3. Decoding the QR Code

**Figure 18:**
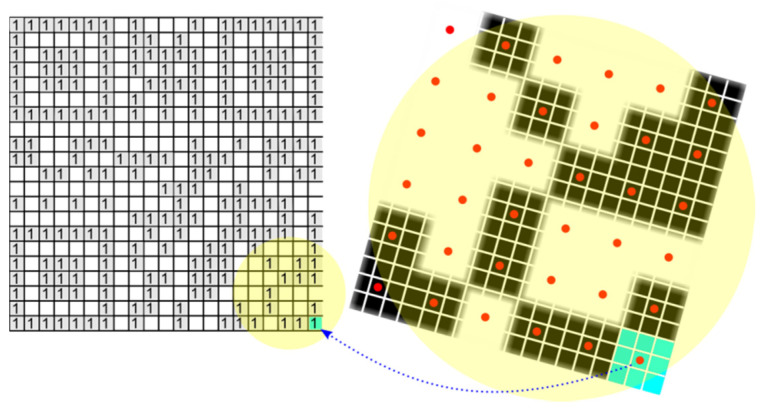
Mapping of the QR Code into the binary matrix.

To successfully decode a QR Code, the precise location of all four corner points which form the bounding quadrilateral, must be done. Once perspective transformation is set up, we can map image points from this quadrilateral into square binary matrix. Dark modules become binary 1 and light modules become binary 0. This binary matrix is the input of a deterministic process that decodes the data stored in the QR Code. If the QR Code is only perspective distorted, then by applying a perspective transformation we get an image of a square QR Code, where its modules are also squares of the same size (Figure 17). In this case, creating the binary matrix is straightforward because it is enough to take the middle points of the modules, which decide whether the module is dark or light (Figure 18). If the modules are larger, we can also consider the average value of several adjacent points around the middle point. Whether a point is classified as dark or light depends on the local threshold value. We divide the whole area of the QR Code into 9 tiles (3 × 3) and for each tile we calculate its threshold value as the grayscale intensity average. Then the local threshold value is determined by bilinear interpolation of the thresholds of adjacent tiles.

**Figure 19:**
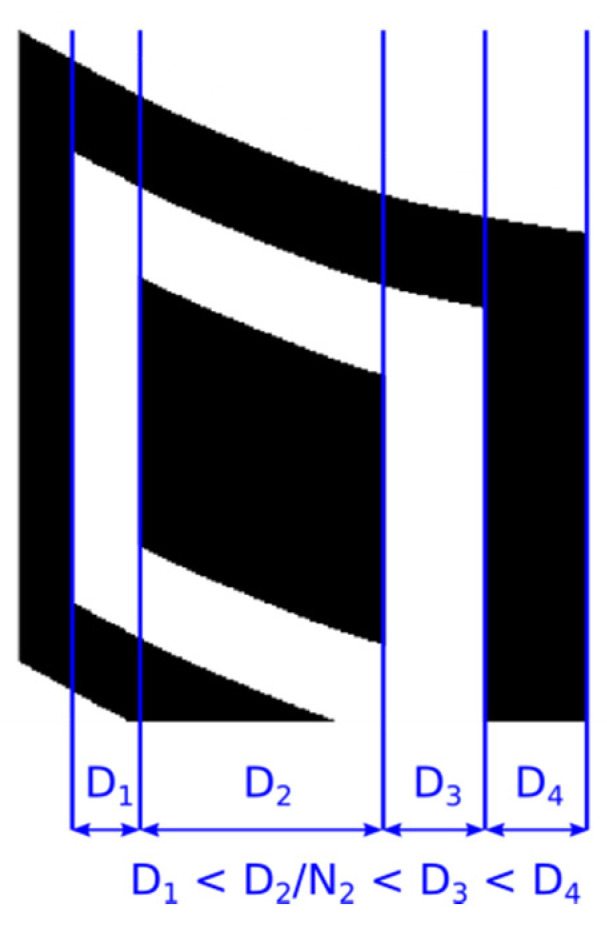
Variable-sized modules.

If the deformation of the QR Code cannot be sufficiently restored only by inverse perspective transformation, for example if a cylindrical distortion is added to the perspective distortion, then the module sizes of the QR Code are not constant (Figure 19). In this case we have to adapt the positions of the middle points to the changing sizes of the modules. Again, we can apply the principle of edge projections when local maxima determine the edges of the modules and distances between two adjacent local maxima gives the size of the current module in the examined row or column. The middle points are then placed in the centre between adjacent local maxima. In the results we refer to this technique as “Dynamic grid”.

## 5. Results

**Figure 20:**
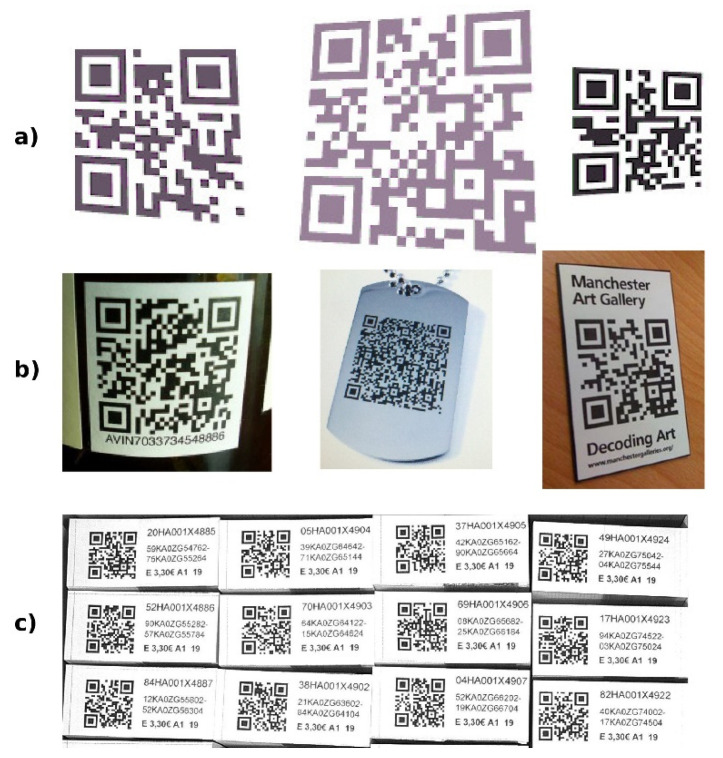
Testing samples: (**a**) synthetic; (**b**) Internet; (**c**) manufacturing.

The method mentioned above was tested on a test set consisting of 286 samples of QR Codes. The testing set contained 20 synthetic QR Codes of various sizes and rotations, 86 QR Codes from Internet images and 180 QR Codes from a specific manufacturing environment. The samples of the test codes are in Figure 20.

**Figure 21:**
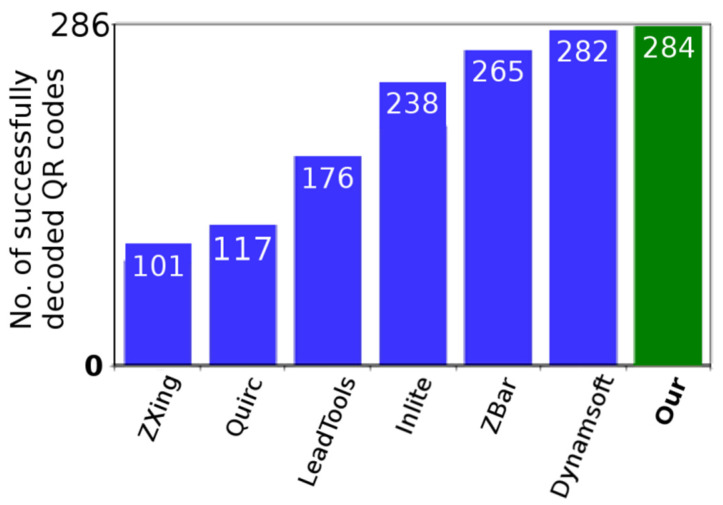
Our results compared with competitive solutions.

In Table 1 and Figure 21 there are our results compared against competitive QR Code decoding software (open-source and also commercial). In the table, there are the numbers of successfully recognized/decoded QR Codes out of a total of 286 codes.

Our method successfully localized all QR Codes, but failed to decode one sample. This sample was a QR Code placed on a bottle, so that the QR Code was perspective- and also cylindrically distorted. How to handle this kind of combined distortion is a challenge for our future research. Lay et al. [36] proposed a parametric cylindrical surface model for rectification of QR Codes printed on cylinders.

As the most of the competitive QR Code decoders are closed source solutions we cannot compare their and our detection algorithms in detail. For this reason, we have performed black-box testing only (using default settings). There does not exists standardized published QR Code testing datasets to compare the results of our method against the results of other previously published methods. Our testing dataset is published online together with this article under “Supplementary Material”.

In Table 2 is compared time consumption of individual solutions depending on the resolution of the input image and the number of QR Codes in one image (the table includes only open-source software that we could test on the same hardware with CPU i5-4590 3.3GHz; our solution was implemented in Free Pascal without using any optimized computer vision libraries).

## 6. Conclusions

We have proposed and tested a precise and fast method for the location of perspective-distorted 2D QR Codes in arbitrary images under various lighting conditions. This method is suitable for localization of single or multiple QR Codes in low-resolution images as well as for real time processing. The proposed methods use typical position detection patterns of QR Codes so-called finder patterns to identify three corners of QR Codes in an image. For distorted QR Codes perspective transformation must be set-up. The optimal position of the fourth corner of the QR Code is determined by analyzing the direction of horizontal and vertical edges and by maximizing the standard deviation of horizontal and vertical projections of these edges. Prerequisites of our method are the existence of intact finder patterns and quiet zones around a QR Code.

The novelty of our method lies in the way the bounding box of a QR Code is determined, especially for perspective-distorted QR Codes and how variable-sized modules are handled.

This method was validated on the testing set consisting of synthetic and also real world samples and it was compared with competitive solutions. The experimental results show that our method has a great detection rate. Unlike other articles, we consider a QR Code to be successfully recognized only if it is also decoded, not just localized. Precise localization is a necessary but not sufficient condition for successful decoding.

**Figure 22:**
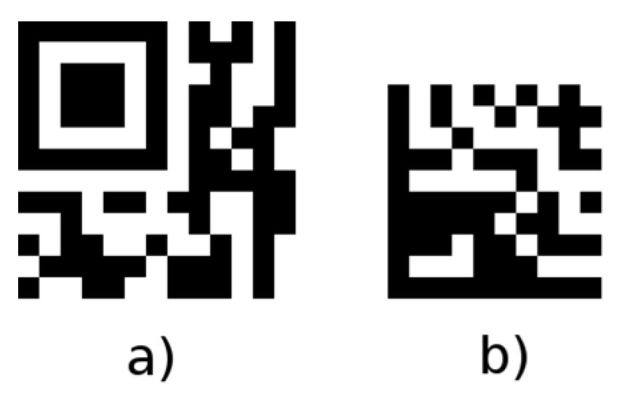
Other types of 2D codes: (**a**) micro QR Code; (**b**) data matrix code.

In the case where it is necessary to place 2D barcodes on a very small area, it is possible to consider the use of a micro QR Code or data matrix code [37] (Figure 22).

## Figures and Tables

**Table 1:** Our results compared with competitive solutions.

Software	Samples (a)	Samples (b)	Samples (c)
Google ZXing (open-source) [30]	1	70	30
Quirc (open-source) [31]	12	66	39
LEADTOOLS QR Code SDK [32]	7	69	100
Inlite Barcode Reader SDK [33]	18	79	141
ZBar (open-source) [34]	20	78	167
Dynamsoft Barcode Reader SDK [35]	20	84	178
**Our—Alternative 1.A, Dynamic grid**	**20**	**85**	**180**
**Our—Alternative 2.A, Dynamic grid**	**20**	**85**	**179**

**Table 2:** Approximate time consumption of tested software.

Software	1024 × 768		1296 × 960		2592 × 1920		
	1 code	10 codes	1 code	10 codes	1 code	10 codes	50 codes
Quirc (open-source)	10 ms	34 ms	15 ms	37 ms	66 ms	130 ms	430 ms
ZBar (open-source)	48 ms	96 ms	76 ms	124 ms	338 ms	396 ms	1781 ms
**Our—Alternative 1.A**	17 ms	77 ms	23 ms	83 ms	74 ms	135 ms	426 ms
**Our—Alternative 2.A**	17 ms	79 ms	22 ms	83 ms	69 ms	129 ms	414 ms

## Supplementary Materials

The following are available online at https://www.mdpi.com/2313-433X/6/7/67/s1, one ZIP file contains images of testing dataset of QR Codes used to test our proposed algorithms.
