# Supplementary figures and images for: Identification of QR Code Perspective Distortion Based on Edge Directions and Edge Projections Analysis
Source: J Imaging. 2020 Jul 10;6(7):67. doi: 10.3390/jimaging6070067 (PMC8321072; doi:10.3390/jimaging6070067)

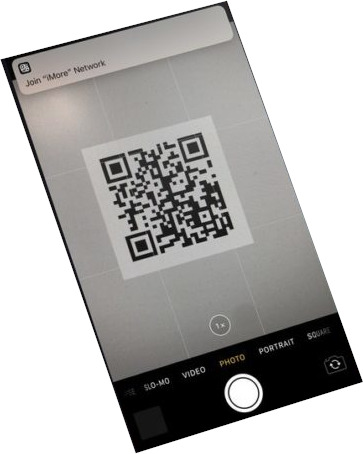

Supplement: Supplementary file 1 [file jimaging-06-00067-s001.zip › QR2v.jpg]

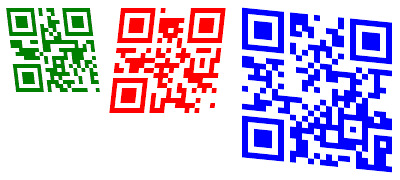

Supplement: Supplementary file 1 [file jimaging-06-00067-s001.zip › QR3l.jpg]

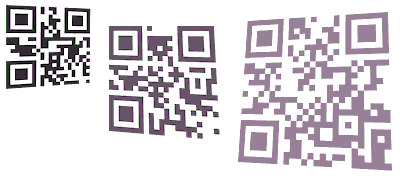

Supplement: Supplementary file 1 [file jimaging-06-00067-s001.zip › QR3m.jpg]

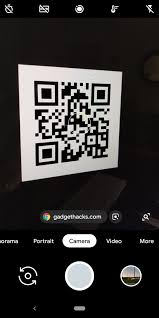

Supplement: Supplementary file 1 [file jimaging-06-00067-s001.zip › QR3n.jpg]

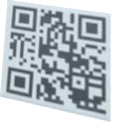

Supplement: Supplementary file 1 [file jimaging-06-00067-s001.zip › QR3o.jpg]

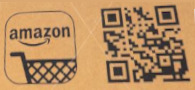

Supplement: Supplementary file 1 [file jimaging-06-00067-s001.zip › QR3p.jpg]

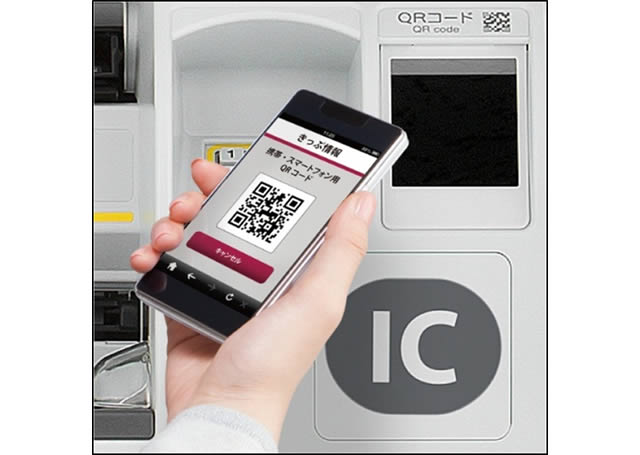

Supplement: Supplementary file 1 [file jimaging-06-00067-s001.zip › QR3k.jpg]

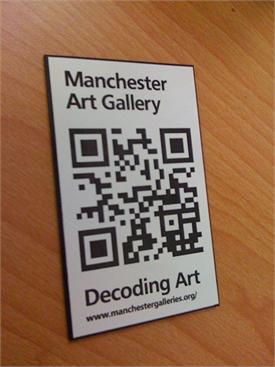

Supplement: Supplementary file 1 [file jimaging-06-00067-s001.zip › QR3j.jpg]

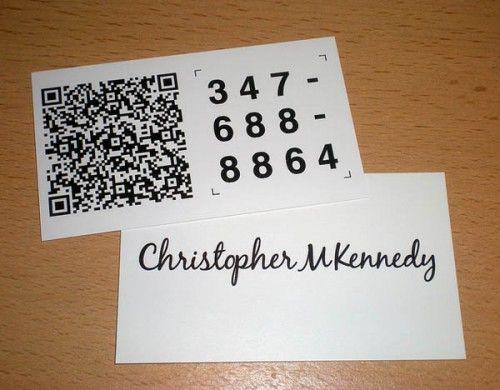

Supplement: Supplementary file 1 [file jimaging-06-00067-s001.zip › QR3i.jpg]

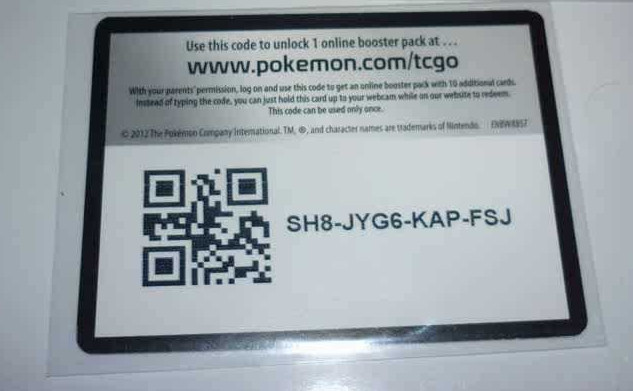

Supplement: Supplementary file 1 [file jimaging-06-00067-s001.zip › QR3h.jpg]

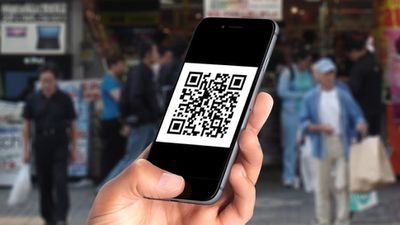

Supplement: Supplementary file 1 [file jimaging-06-00067-s001.zip › QR3g.jpg]

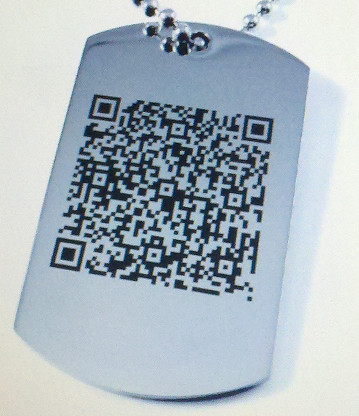

Supplement: Supplementary file 1 [file jimaging-06-00067-s001.zip › QR3f.jpg]

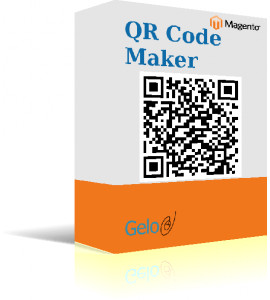

Supplement: Supplementary file 1 [file jimaging-06-00067-s001.zip › QR3e.jpg]

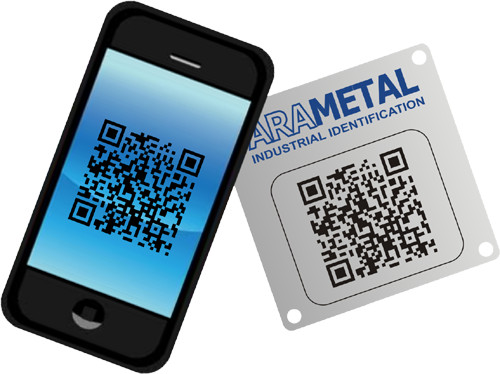

Supplement: Supplementary file 1 [file jimaging-06-00067-s001.zip › QR2u.jpg]

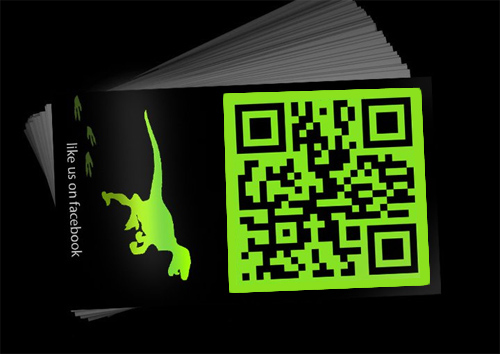

Supplement: Supplementary file 1 [file jimaging-06-00067-s001.zip › QR2t.jpg]

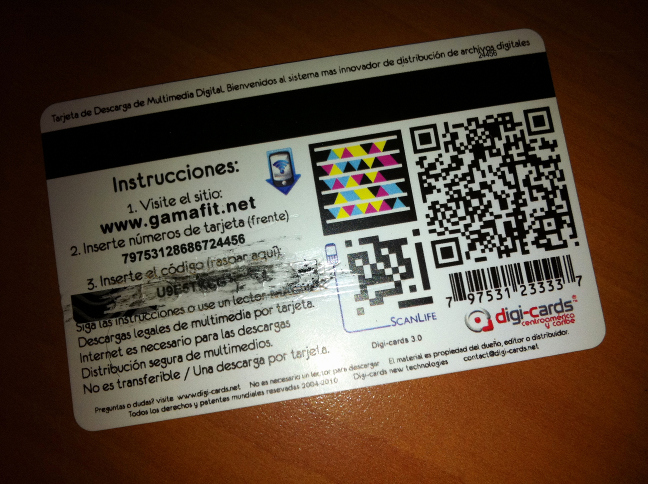

Supplement: Supplementary file 1 [file jimaging-06-00067-s001.zip › QR3d.jpg]

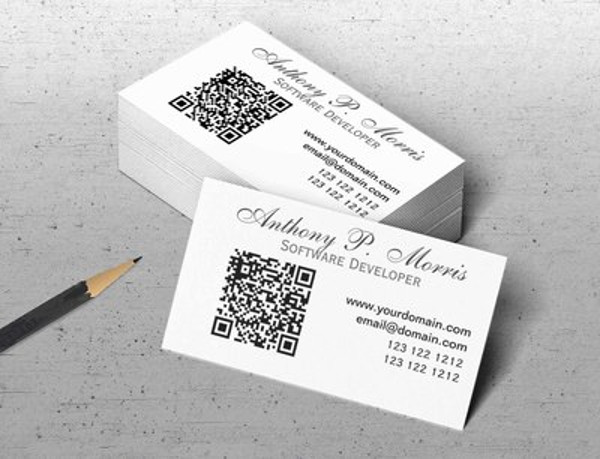

Supplement: Supplementary file 1 [file jimaging-06-00067-s001.zip › QR2s.jpg]

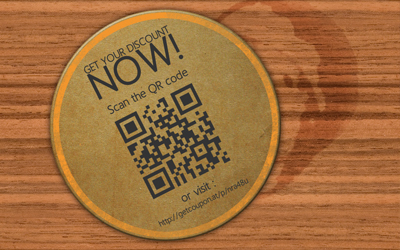

Supplement: Supplementary file 1 [file jimaging-06-00067-s001.zip › QR2r.jpg]

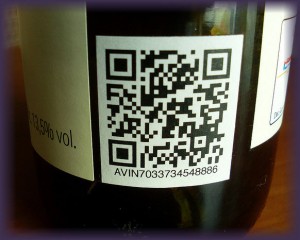

Supplement: Supplementary file 1 [file jimaging-06-00067-s001.zip › QR3c.jpg]

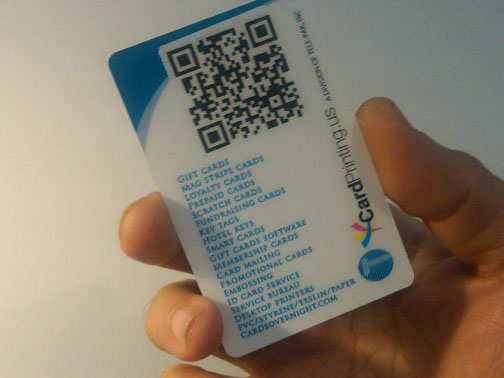

Supplement: Supplementary file 1 [file jimaging-06-00067-s001.zip › QR2q.jpg]

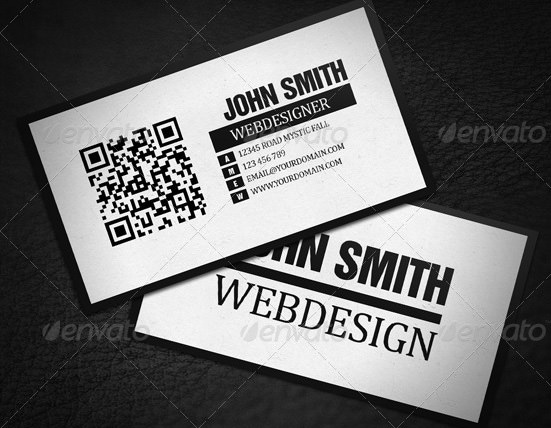

Supplement: Supplementary file 1 [file jimaging-06-00067-s001.zip › QR2p.jpg]

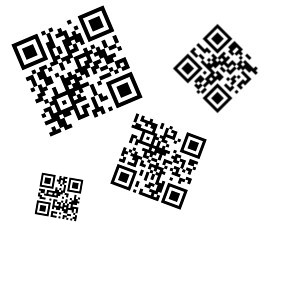

Supplement: Supplementary file 1 [file jimaging-06-00067-s001.zip › QR1b.jpg]

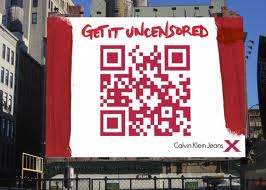

Supplement: Supplementary file 1 [file jimaging-06-00067-s001.zip › QR2o.jpg]

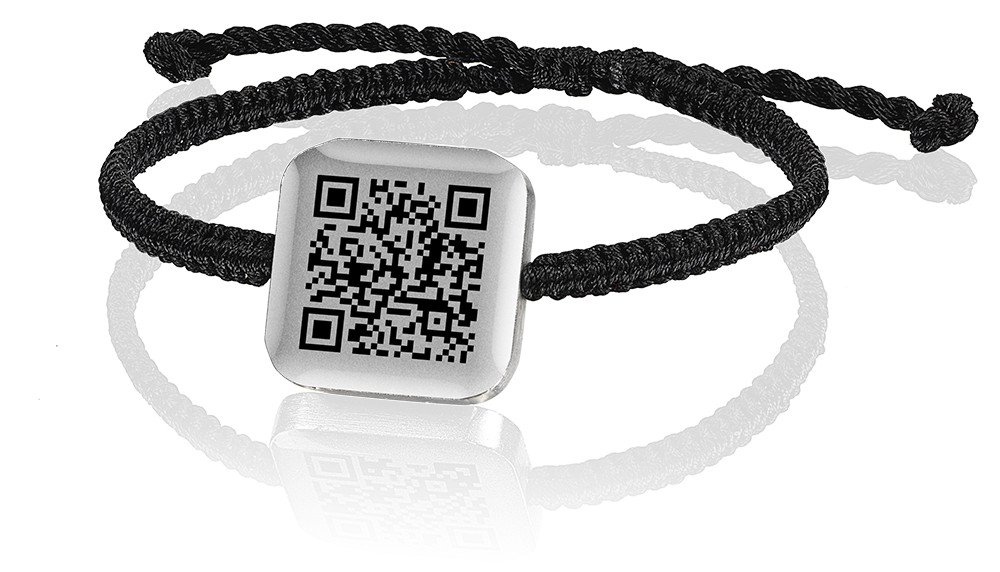

Supplement: Supplementary file 1 [file jimaging-06-00067-s001.zip › QR2n.jpg]

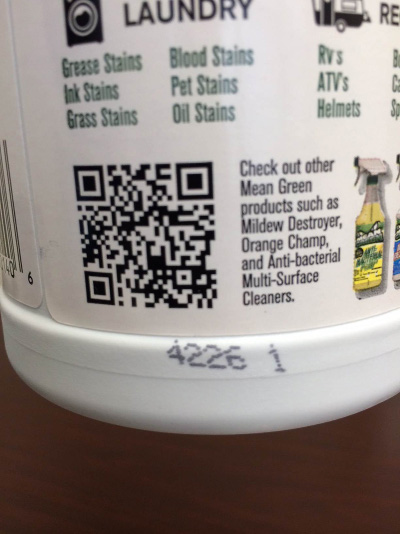

Supplement: Supplementary file 1 [file jimaging-06-00067-s001.zip › QR3b.jpg]

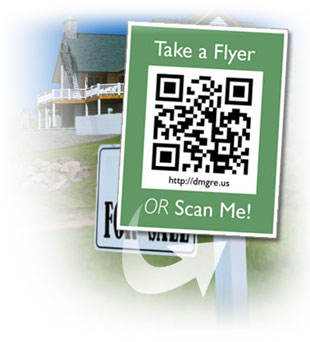

Supplement: Supplementary file 1 [file jimaging-06-00067-s001.zip › QR2l.jpg]

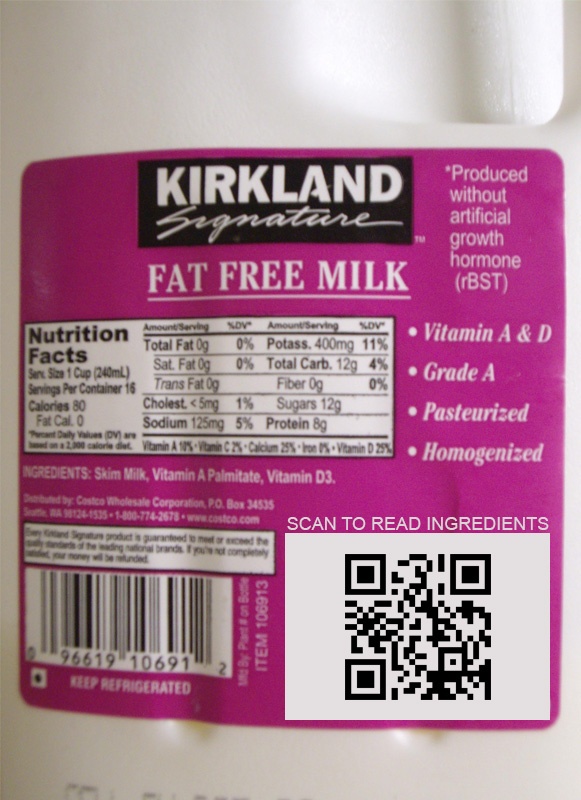

Supplement: Supplementary file 1 [file jimaging-06-00067-s001.zip › QR2k.jpg]

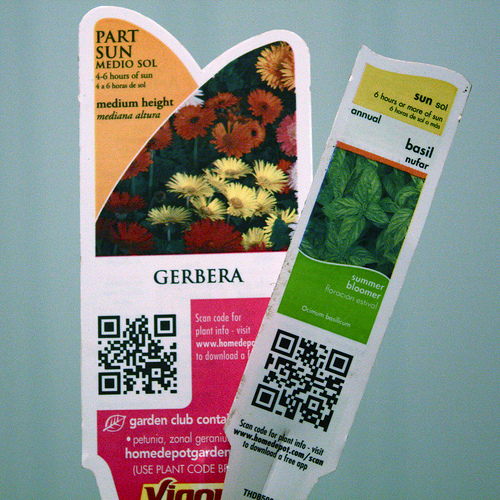

Supplement: Supplementary file 1 [file jimaging-06-00067-s001.zip › QR2m.jpg]

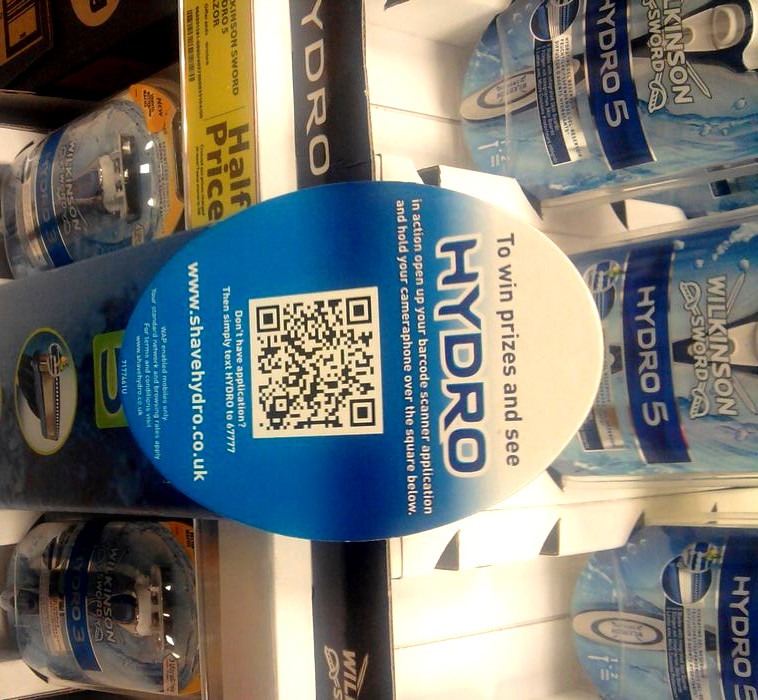

Supplement: Supplementary file 1 [file jimaging-06-00067-s001.zip › QR3a.jpg]

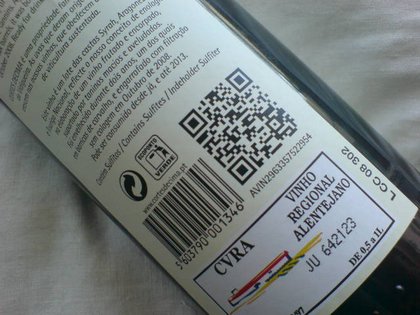

Supplement: Supplementary file 1 [file jimaging-06-00067-s001.zip › QR2j.jpg]

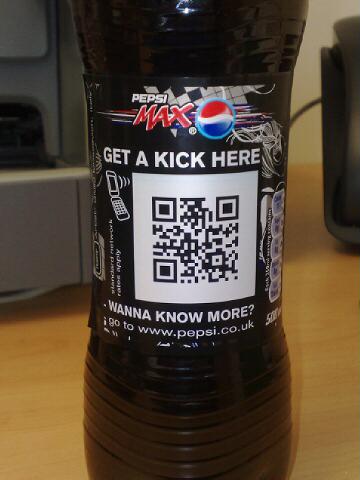

Supplement: Supplementary file 1 [file jimaging-06-00067-s001.zip › QR2i.jpg]

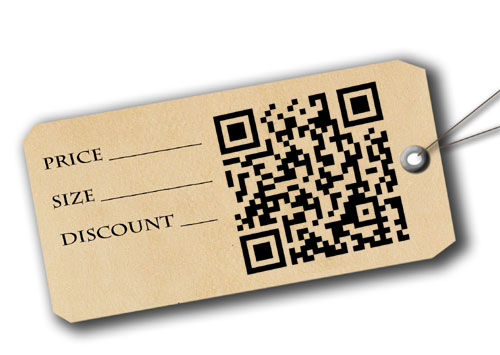

Supplement: Supplementary file 1 [file jimaging-06-00067-s001.zip › QR2h.jpg]

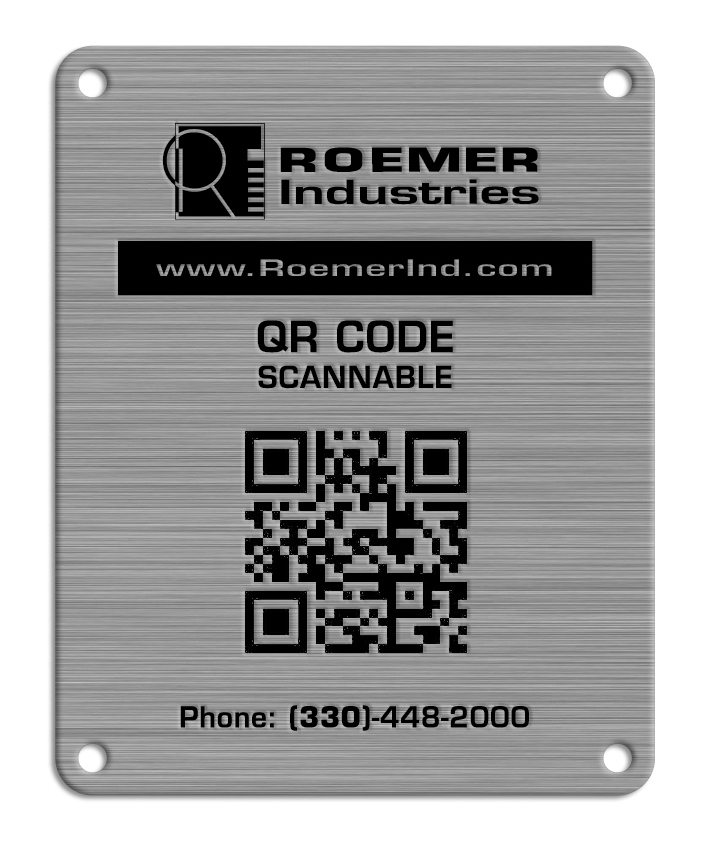

Supplement: Supplementary file 1 [file jimaging-06-00067-s001.zip › QR2g.jpg]

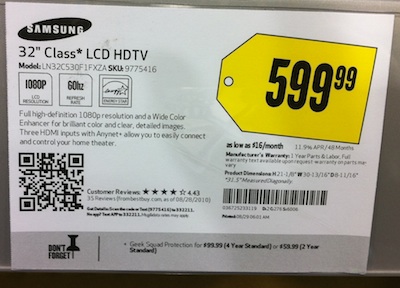

Supplement: Supplementary file 1 [file jimaging-06-00067-s001.zip › QR2f.jpg]

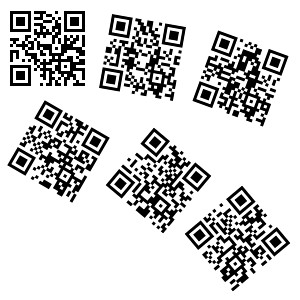

Supplement: Supplementary file 1 [file jimaging-06-00067-s001.zip › QR1c.jpg]

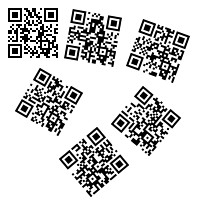

Supplement: Supplementary file 1 [file jimaging-06-00067-s001.zip › QR1d.jpg]

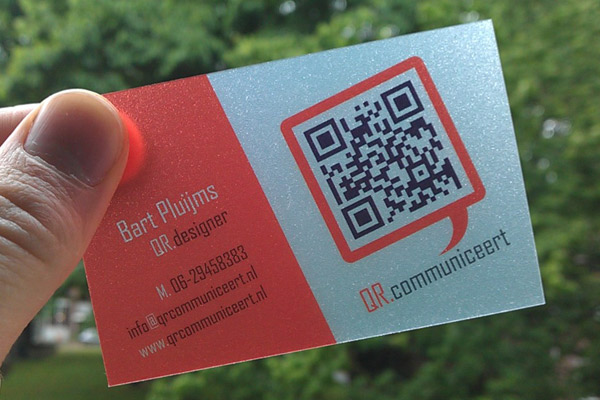

Supplement: Supplementary file 1 [file jimaging-06-00067-s001.zip › QR2e.jpg]

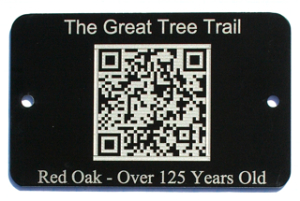

Supplement: Supplementary file 1 [file jimaging-06-00067-s001.zip › QR2d.jpg]

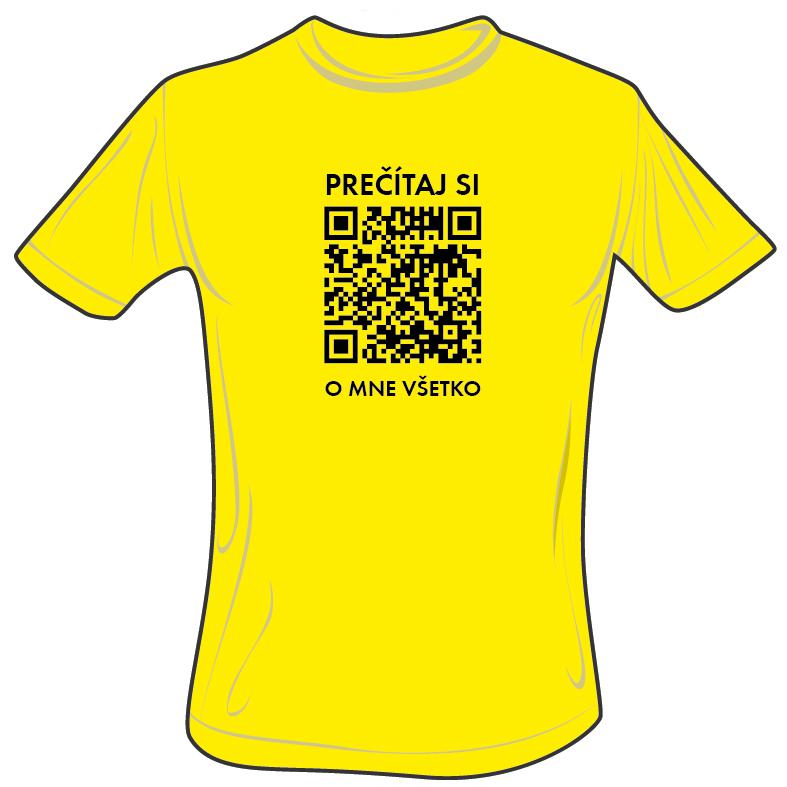

Supplement: Supplementary file 1 [file jimaging-06-00067-s001.zip › QR2c.jpg]

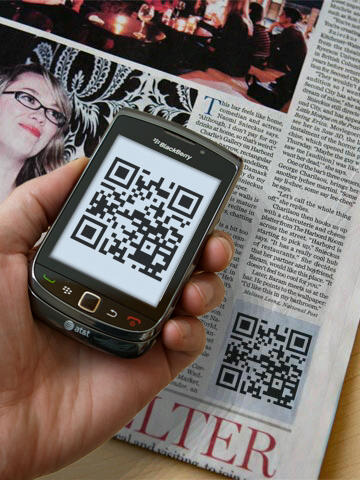

Supplement: Supplementary file 1 [file jimaging-06-00067-s001.zip › QR2b.jpg]

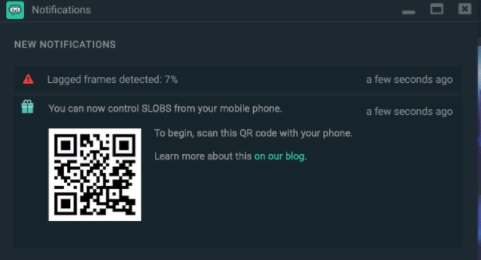

Supplement: Supplementary file 1 [file jimaging-06-00067-s001.zip › QR2a.jpg]

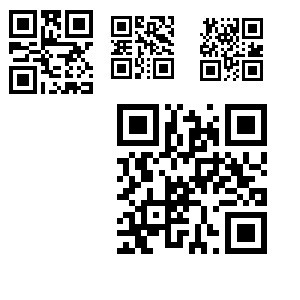

Supplement: Supplementary file 1 [file jimaging-06-00067-s001.zip › QR1a.jpg]

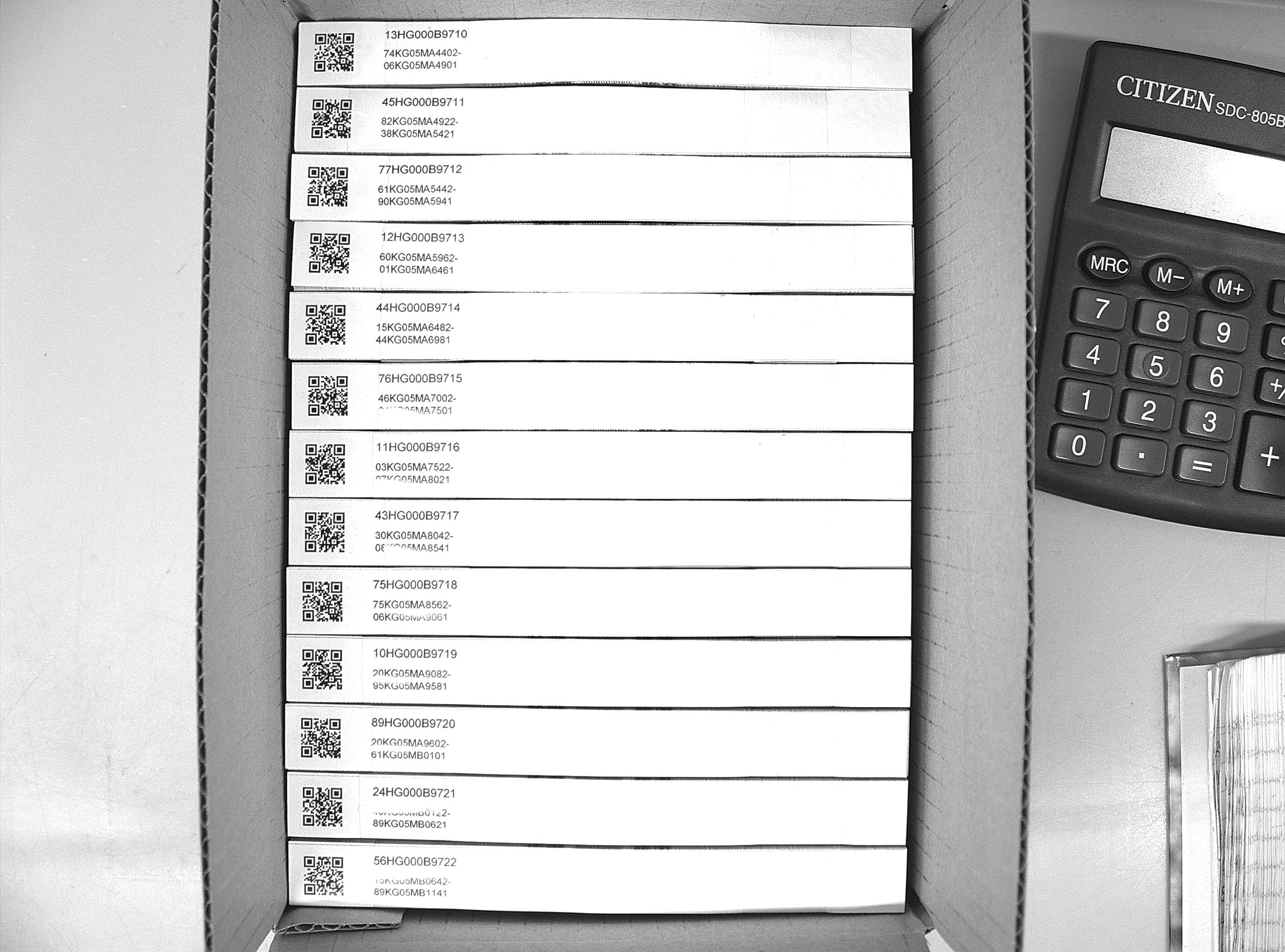

Supplement: Supplementary file 1 [file jimaging-06-00067-s001.zip › QR4d.jpg]

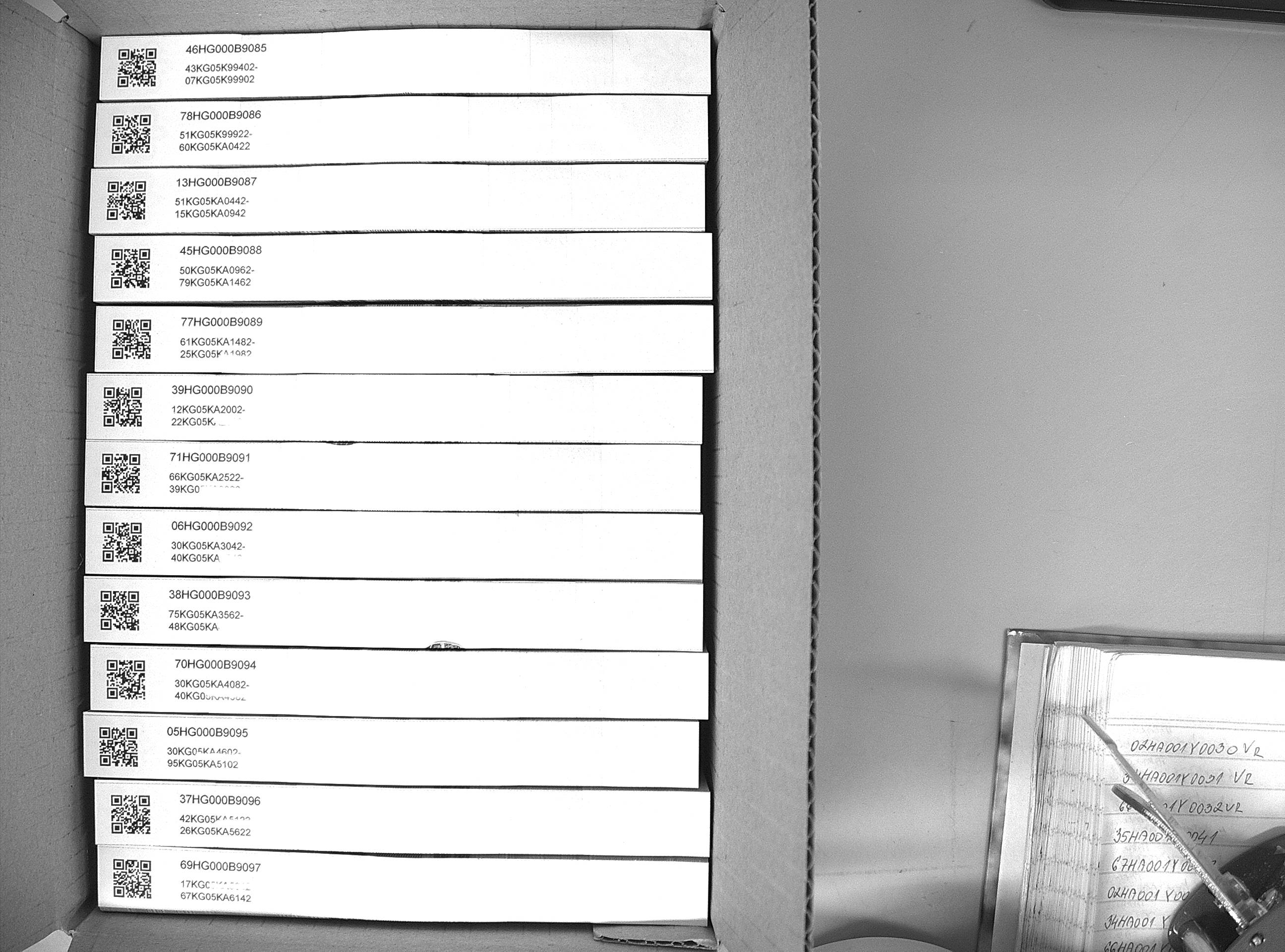

Supplement: Supplementary file 1 [file jimaging-06-00067-s001.zip › QR4c.jpg]

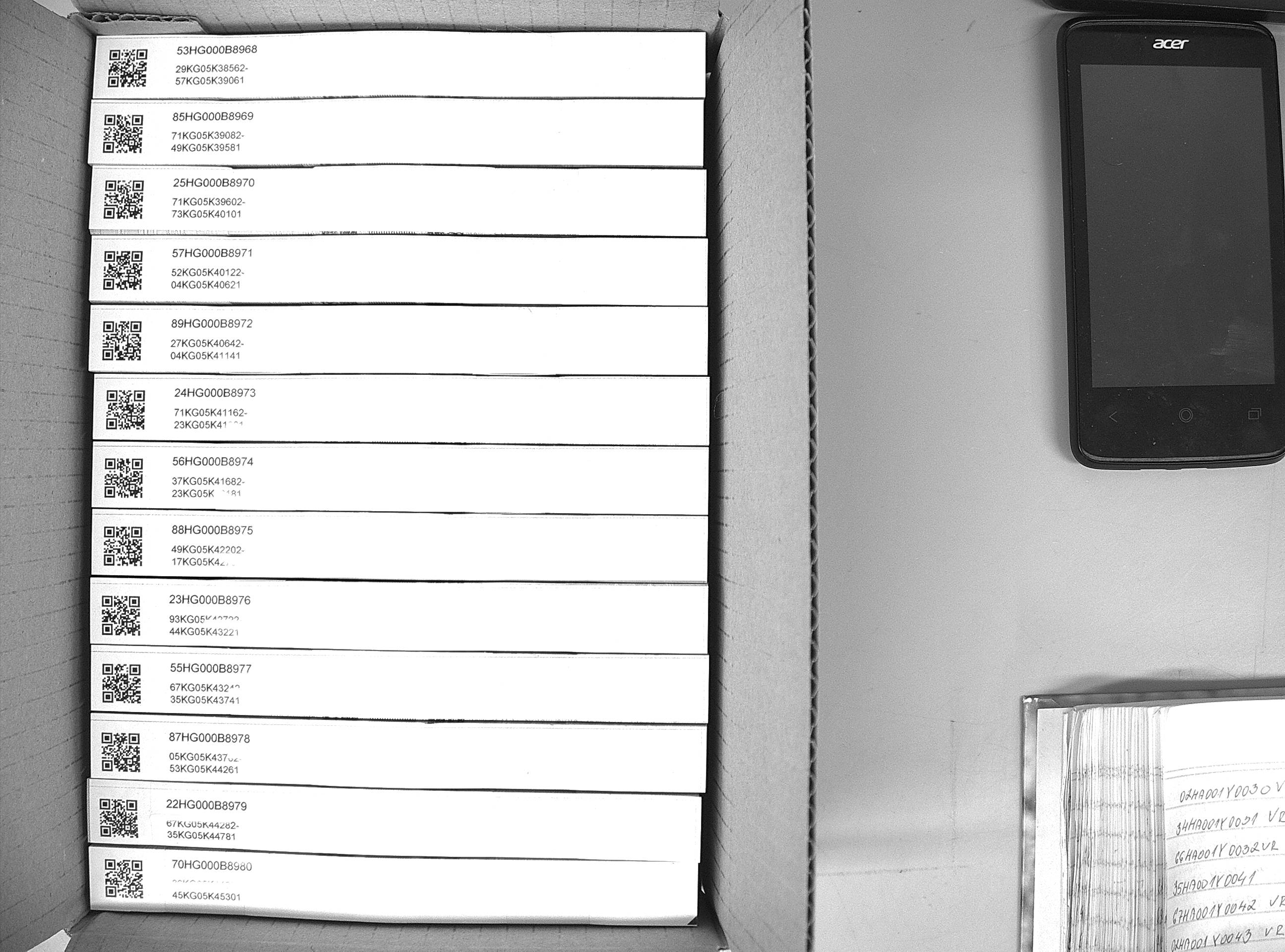

Supplement: Supplementary file 1 [file jimaging-06-00067-s001.zip › QR4a.jpg]

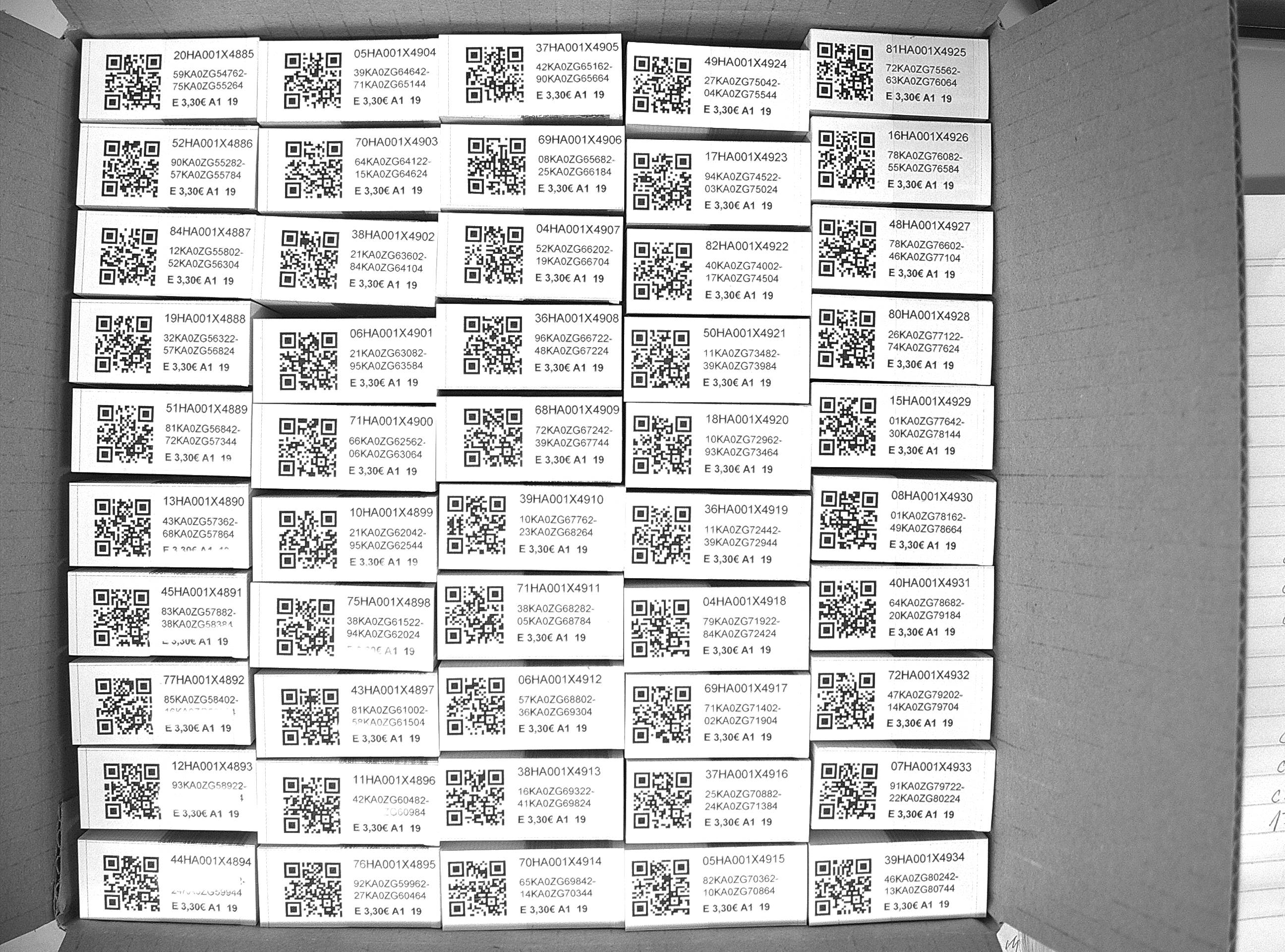

Supplement: Supplementary file 1 [file jimaging-06-00067-s001.zip › QR4b.jpg]

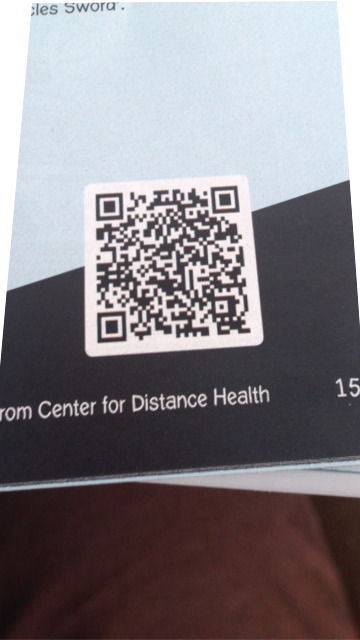

Supplement: Supplementary file 1 [file jimaging-06-00067-s001.zip › QR5q2.jpg]

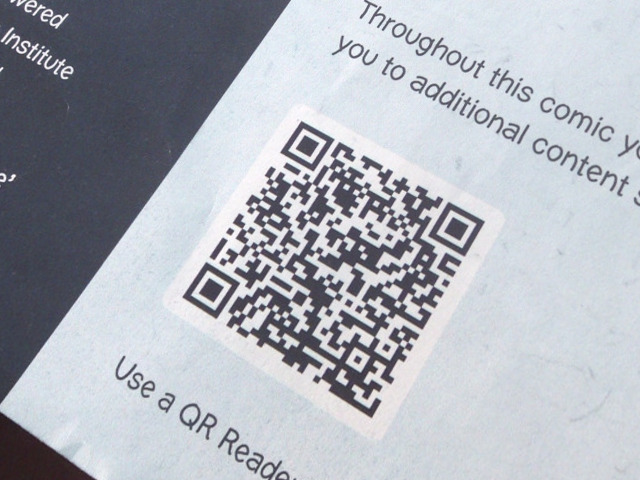

Supplement: Supplementary file 1 [file jimaging-06-00067-s001.zip › QR5a2.jpg]

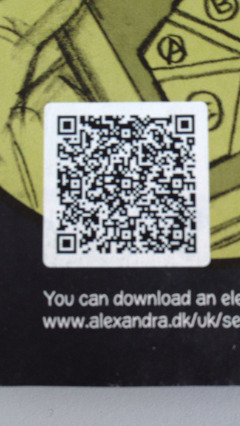

Supplement: Supplementary file 1 [file jimaging-06-00067-s001.zip › QR5o3.jpg]

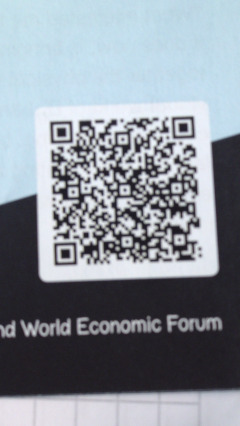

Supplement: Supplementary file 1 [file jimaging-06-00067-s001.zip › QR5l4.jpg]

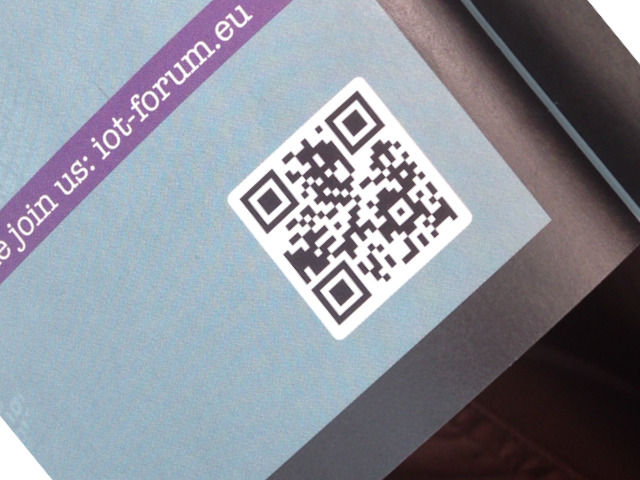

Supplement: Supplementary file 1 [file jimaging-06-00067-s001.zip › QR5n3.jpg]

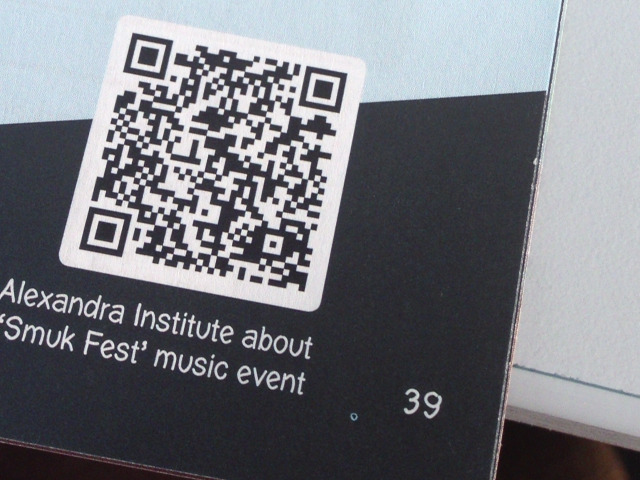

Supplement: Supplementary file 1 [file jimaging-06-00067-s001.zip › QR5b2.jpg]

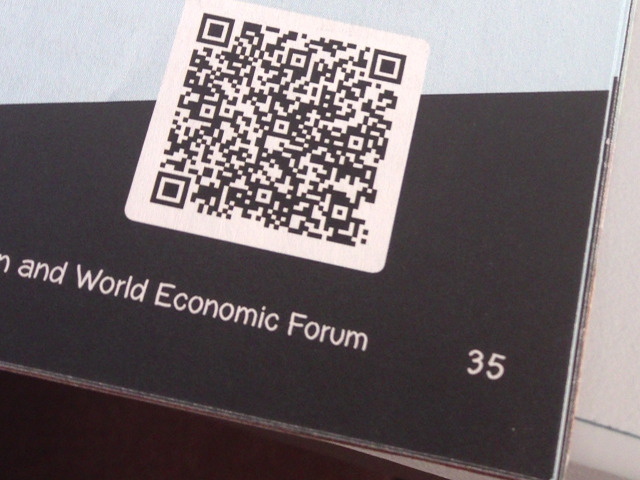

Supplement: Supplementary file 1 [file jimaging-06-00067-s001.zip › QR5l3.jpg]

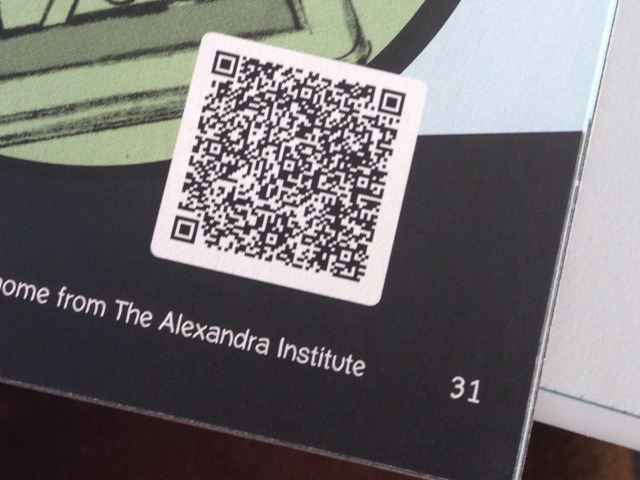

Supplement: Supplementary file 1 [file jimaging-06-00067-s001.zip › QR5p2.jpg]

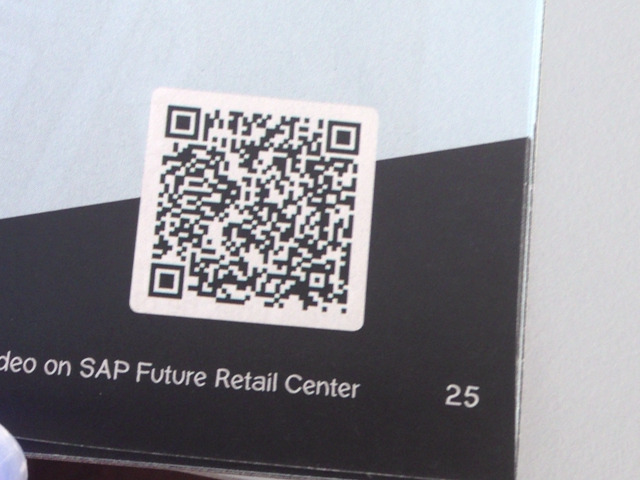

Supplement: Supplementary file 1 [file jimaging-06-00067-s001.zip › QR5i3.jpg]

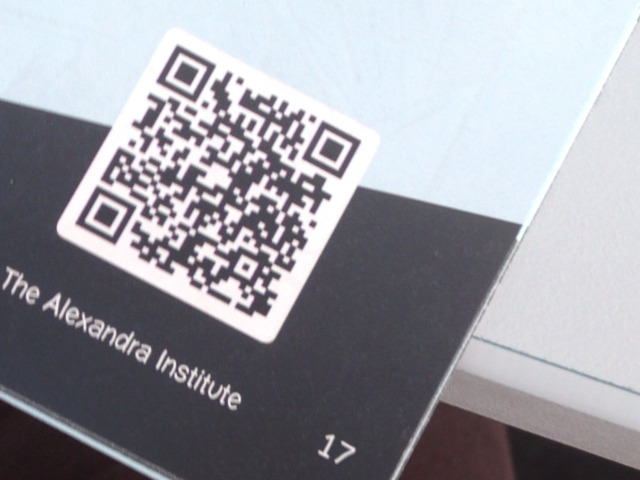

Supplement: Supplementary file 1 [file jimaging-06-00067-s001.zip › QR5g2.jpg]

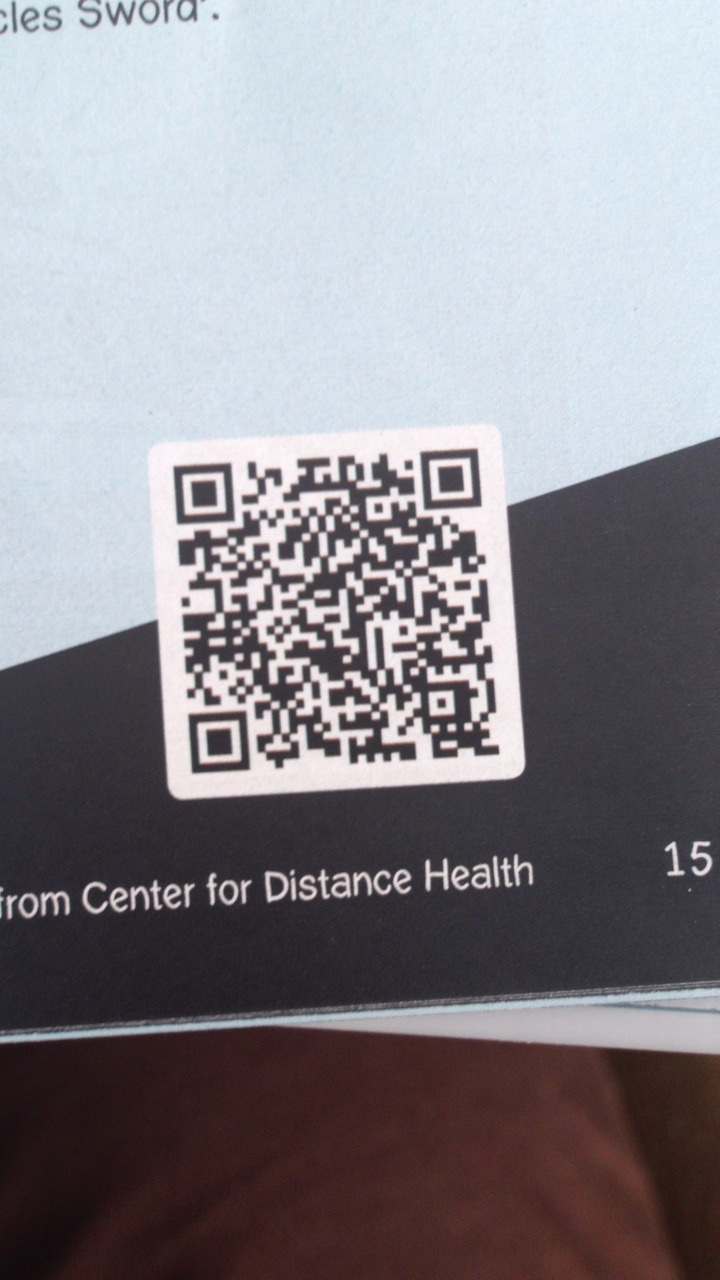

Supplement: Supplementary file 1 [file jimaging-06-00067-s001.zip › QR5q1.jpg]

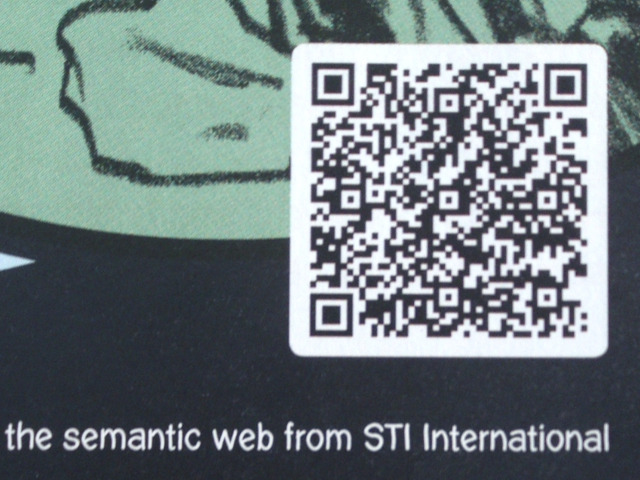

Supplement: Supplementary file 1 [file jimaging-06-00067-s001.zip › QR5e3.jpg]

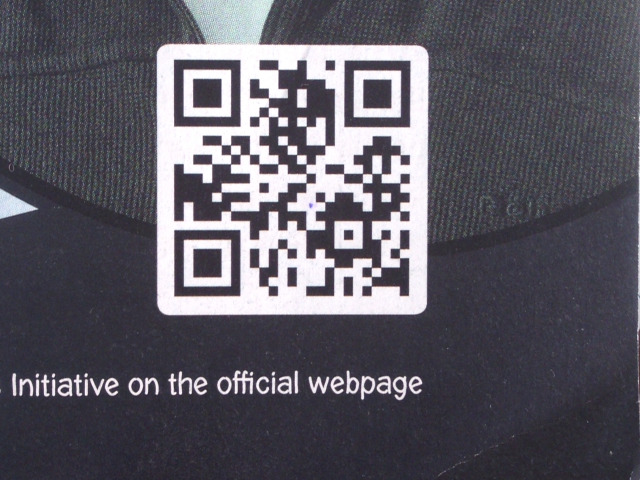

Supplement: Supplementary file 1 [file jimaging-06-00067-s001.zip › QR5n2.jpg]

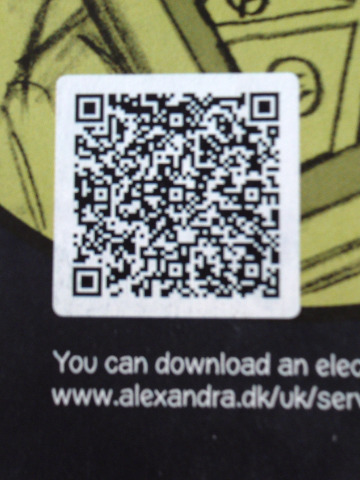

Supplement: Supplementary file 1 [file jimaging-06-00067-s001.zip › QR5o2.jpg]

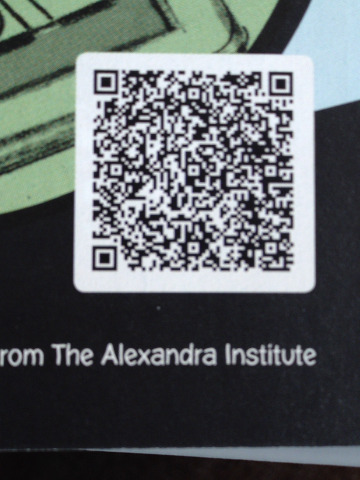

Supplement: Supplementary file 1 [file jimaging-06-00067-s001.zip › QR5p1.jpg]

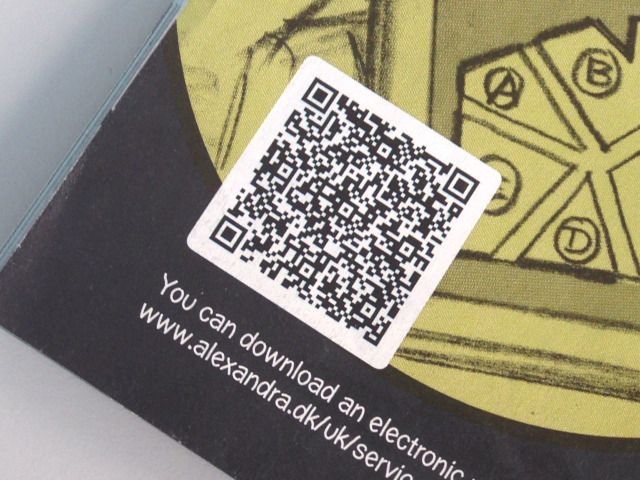

Supplement: Supplementary file 1 [file jimaging-06-00067-s001.zip › QR5o1.jpg]

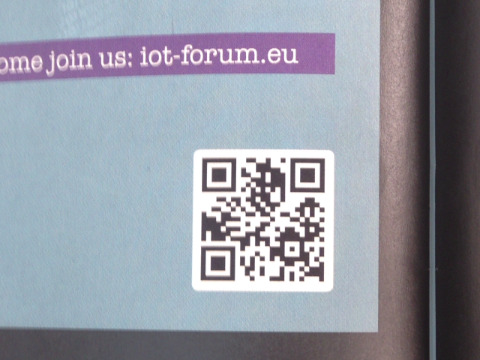

Supplement: Supplementary file 1 [file jimaging-06-00067-s001.zip › QR5n1.jpg]

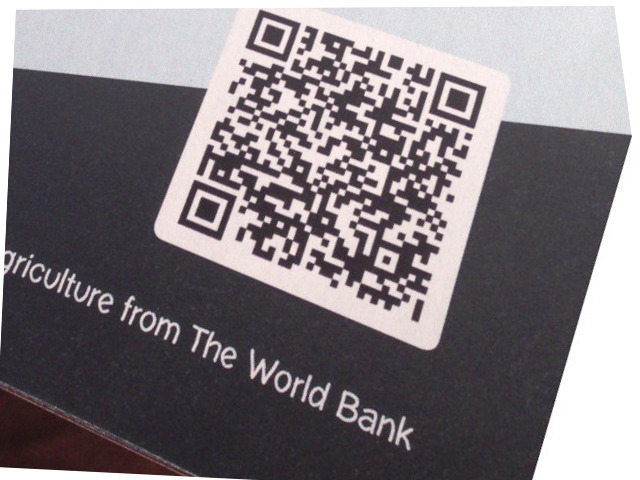

Supplement: Supplementary file 1 [file jimaging-06-00067-s001.zip › QR5m1.jpg]

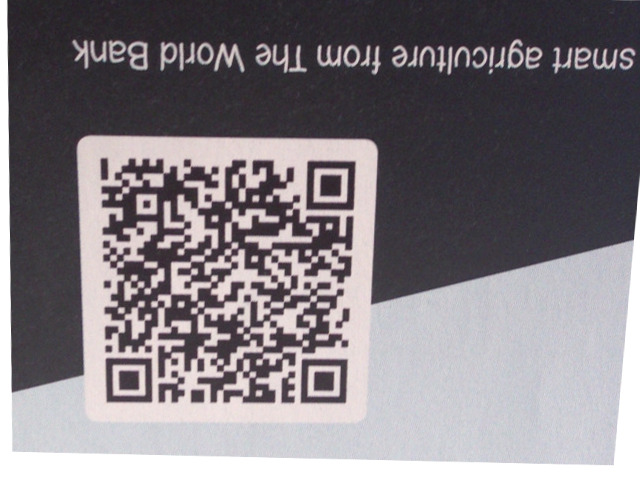

Supplement: Supplementary file 1 [file jimaging-06-00067-s001.zip › QR5m2.jpg]

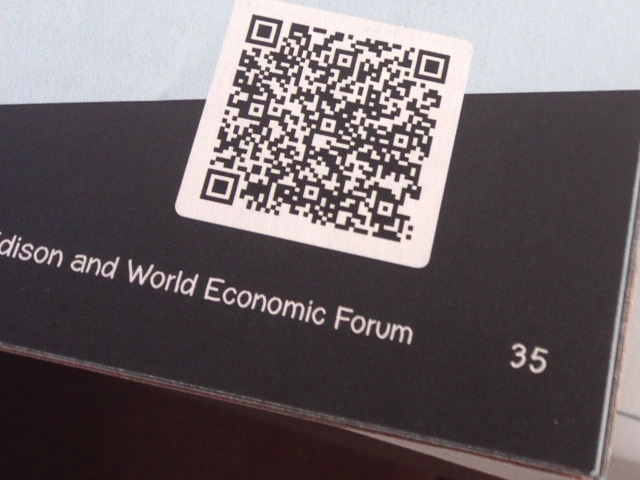

Supplement: Supplementary file 1 [file jimaging-06-00067-s001.zip › QR5l1.jpg]

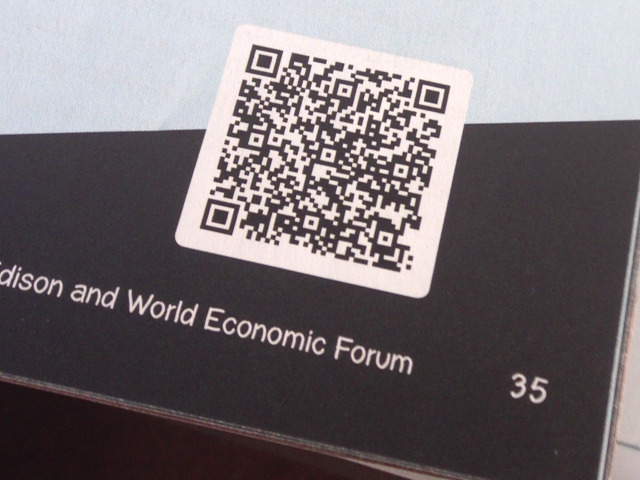

Supplement: Supplementary file 1 [file jimaging-06-00067-s001.zip › QR5l2.jpg]

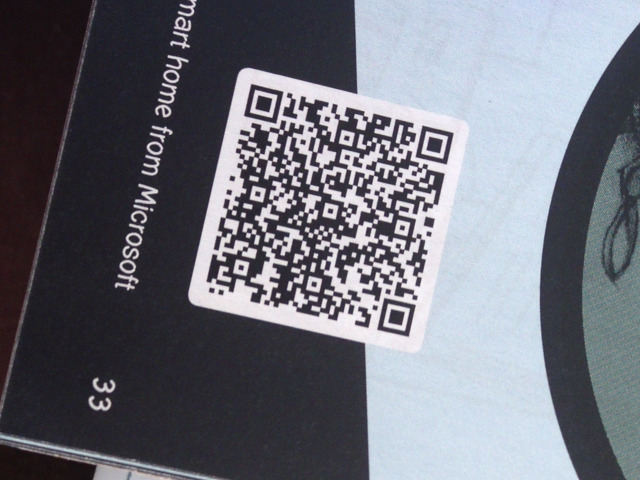

Supplement: Supplementary file 1 [file jimaging-06-00067-s001.zip › QR5k2.jpg]

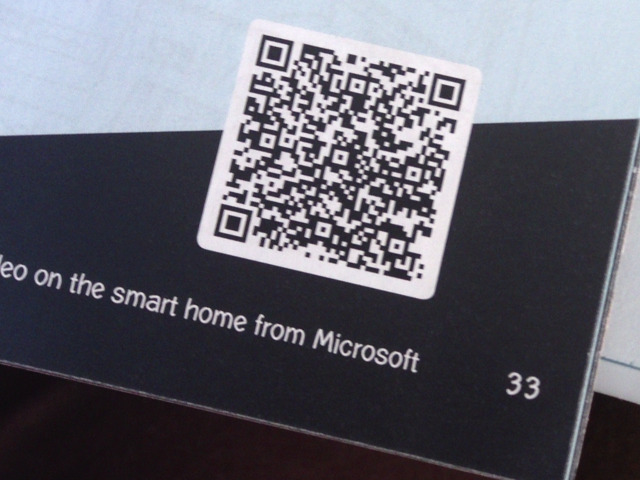

Supplement: Supplementary file 1 [file jimaging-06-00067-s001.zip › QR5k1.jpg]

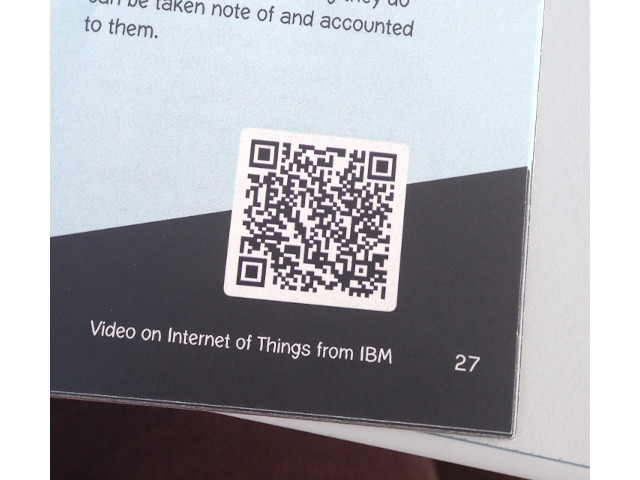

Supplement: Supplementary file 1 [file jimaging-06-00067-s001.zip › QR5j1.jpg]

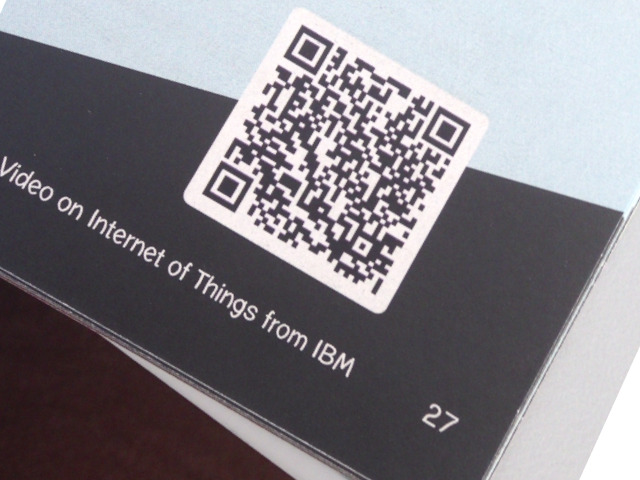

Supplement: Supplementary file 1 [file jimaging-06-00067-s001.zip › QR5j2.jpg]

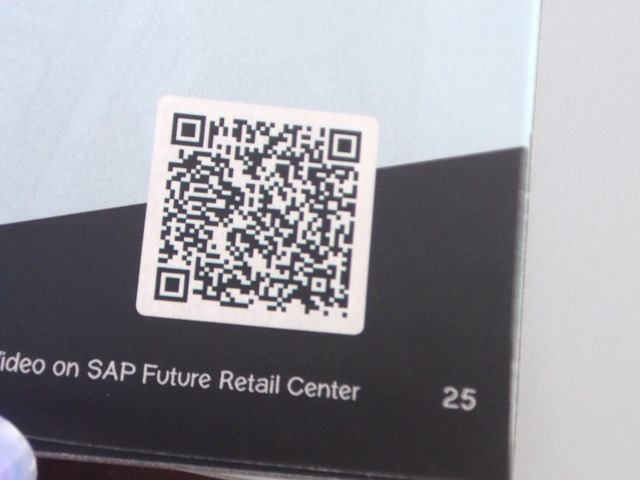

Supplement: Supplementary file 1 [file jimaging-06-00067-s001.zip › QR5i2.jpg]

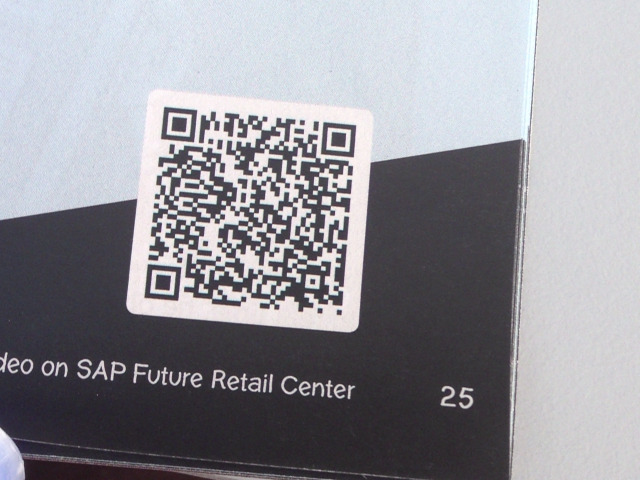

Supplement: Supplementary file 1 [file jimaging-06-00067-s001.zip › QR5i1.jpg]

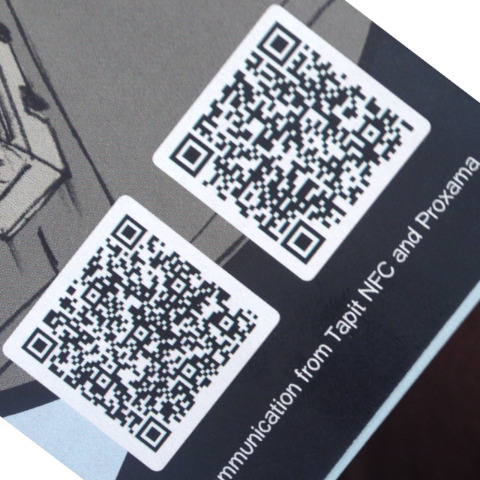

Supplement: Supplementary file 1 [file jimaging-06-00067-s001.zip › QR5h1.jpg]

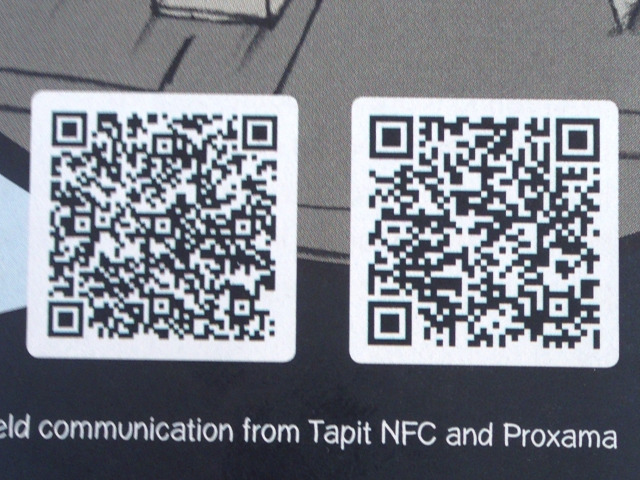

Supplement: Supplementary file 1 [file jimaging-06-00067-s001.zip › QR5h2.jpg]

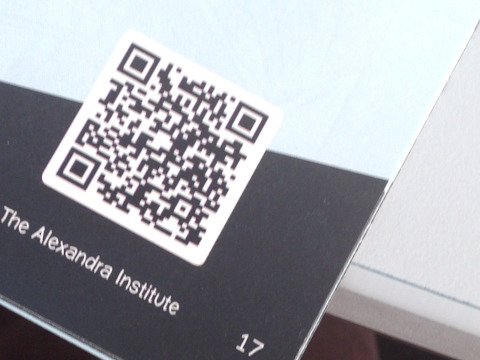

Supplement: Supplementary file 1 [file jimaging-06-00067-s001.zip › QR5g1.jpg]

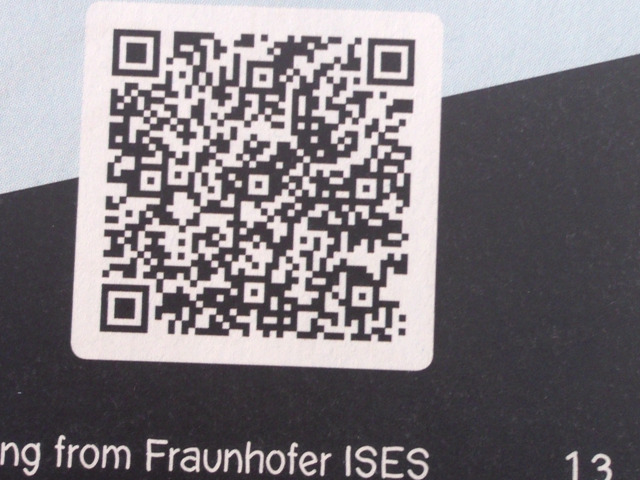

Supplement: Supplementary file 1 [file jimaging-06-00067-s001.zip › QR5f1.jpg]

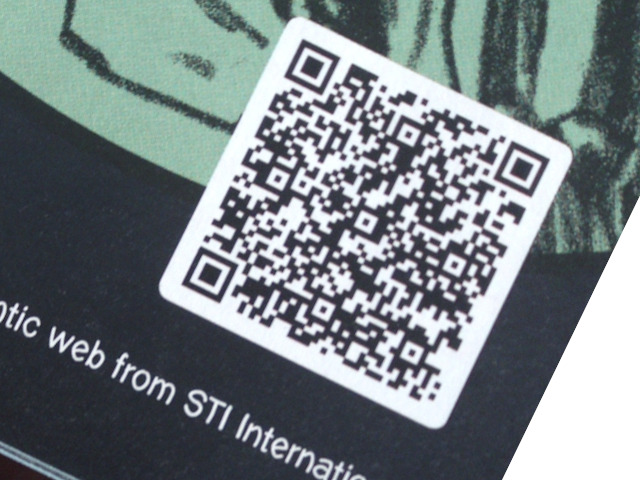

Supplement: Supplementary file 1 [file jimaging-06-00067-s001.zip › QR5e2.jpg]

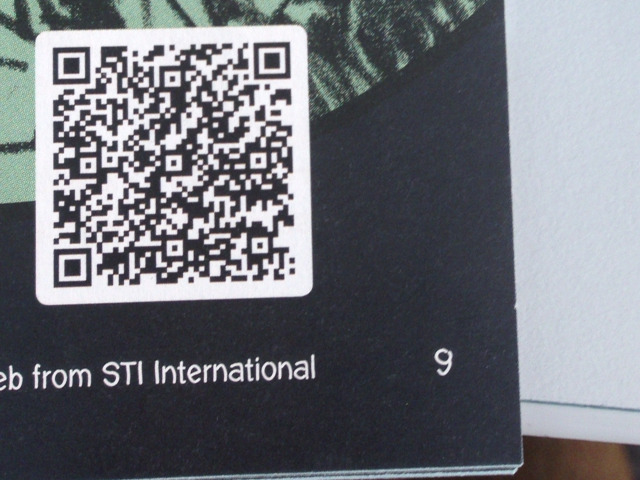

Supplement: Supplementary file 1 [file jimaging-06-00067-s001.zip › QR5e1.jpg]

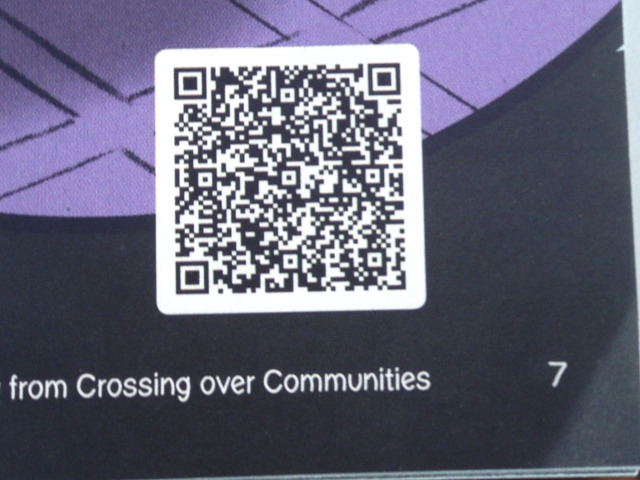

Supplement: Supplementary file 1 [file jimaging-06-00067-s001.zip › QR5d1.jpg]

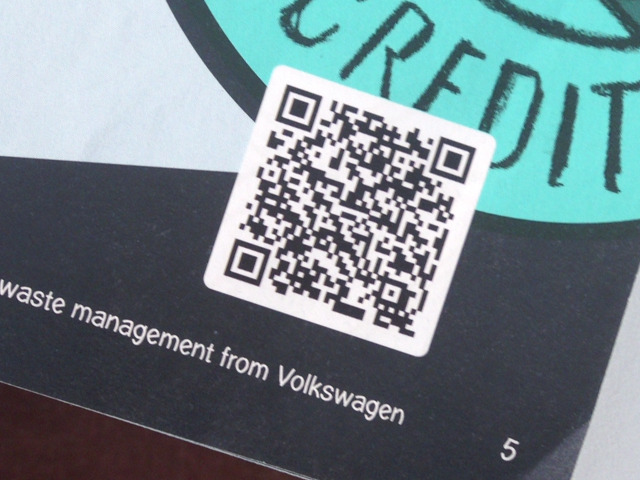

Supplement: Supplementary file 1 [file jimaging-06-00067-s001.zip › QR5c2.jpg]

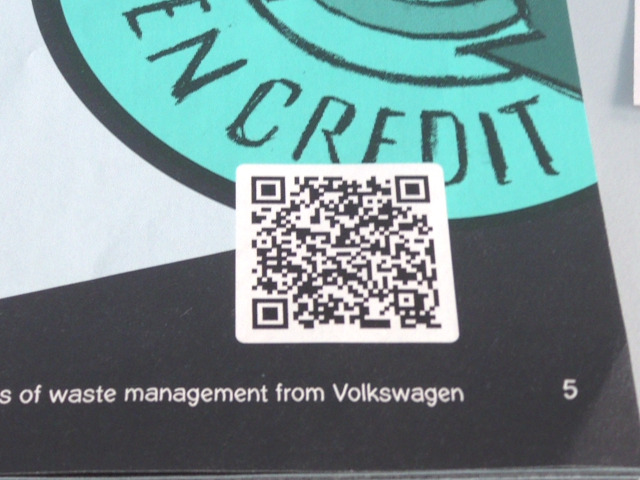

Supplement: Supplementary file 1 [file jimaging-06-00067-s001.zip › QR5c1.jpg]

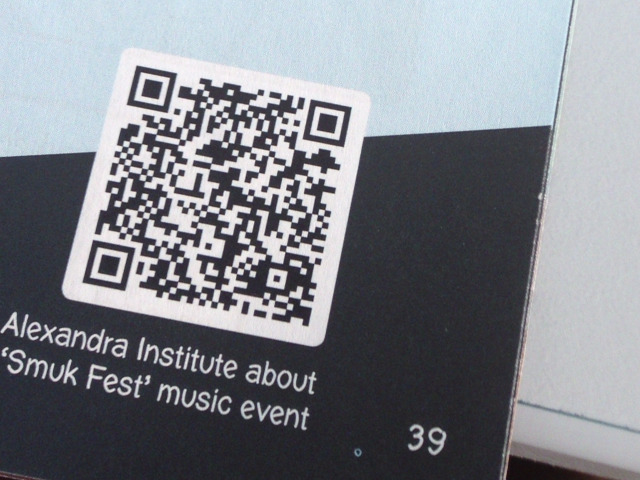

Supplement: Supplementary file 1 [file jimaging-06-00067-s001.zip › QR5b1.jpg]

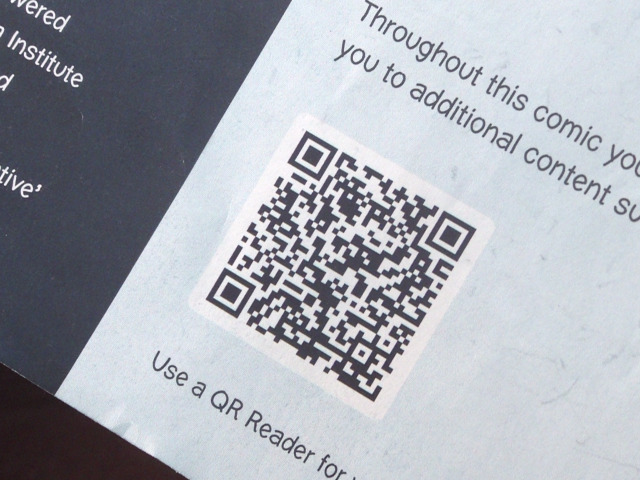

Supplement: Supplementary file 1 [file jimaging-06-00067-s001.zip › QR5a1.jpg]

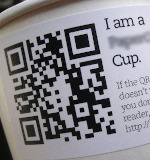

Supplement: Supplementary file 1 [file jimaging-06-00067-s001.zip › QR3q.jpg]

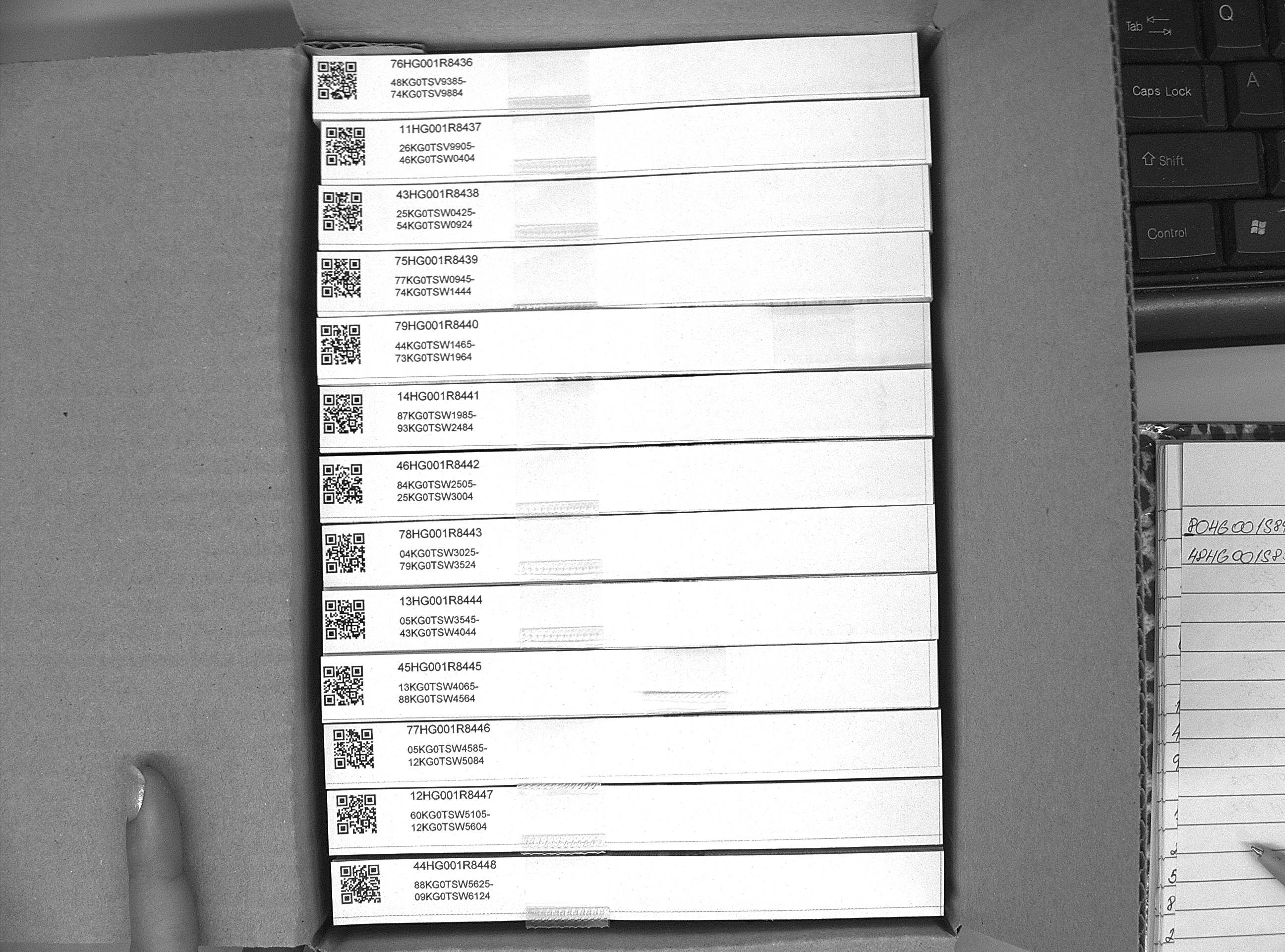

Supplement: Supplementary file 1 [file jimaging-06-00067-s001.zip › QR4e.jpg]

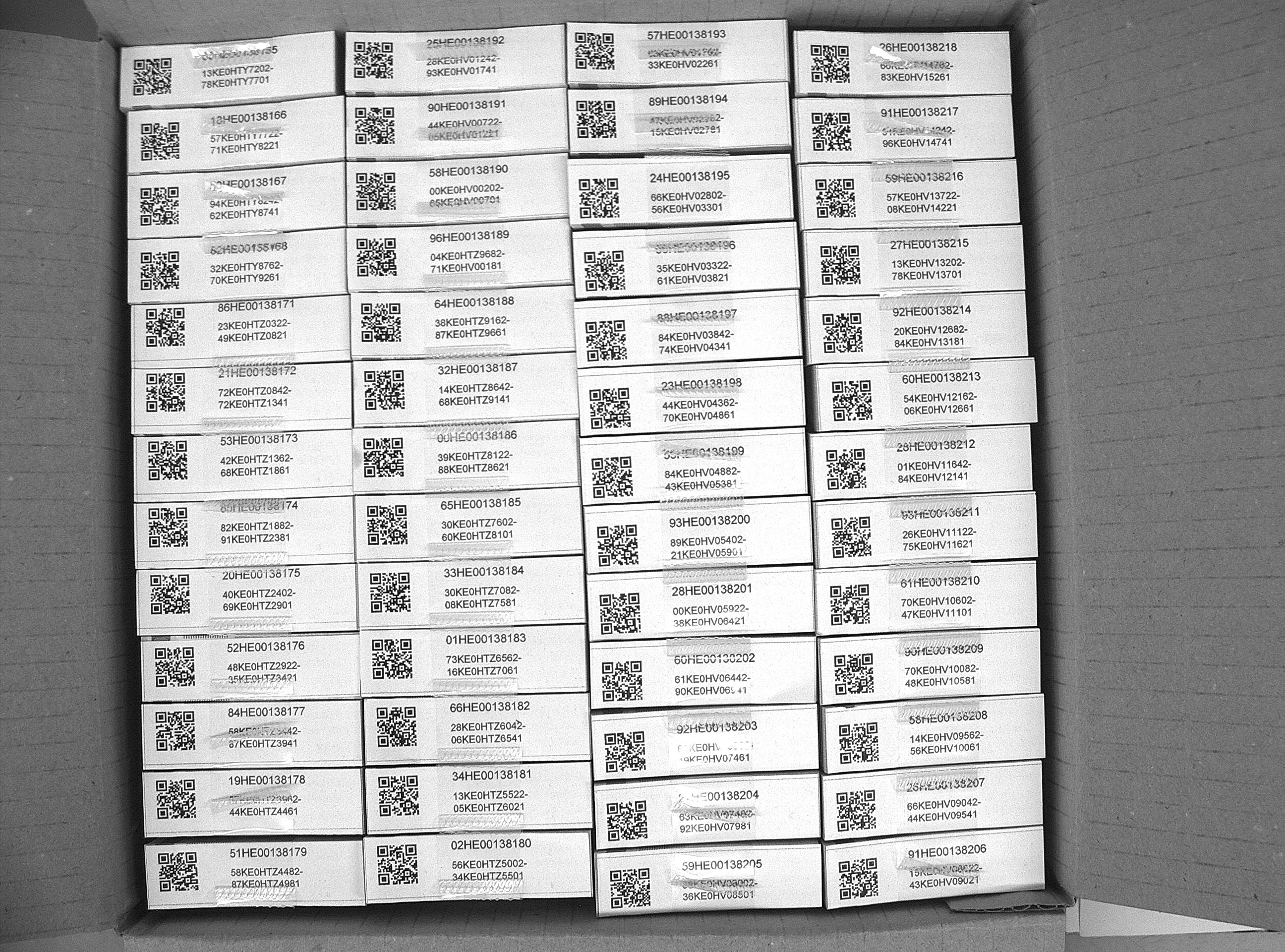

Supplement: Supplementary file 1 [file jimaging-06-00067-s001.zip › QR4g.jpg]

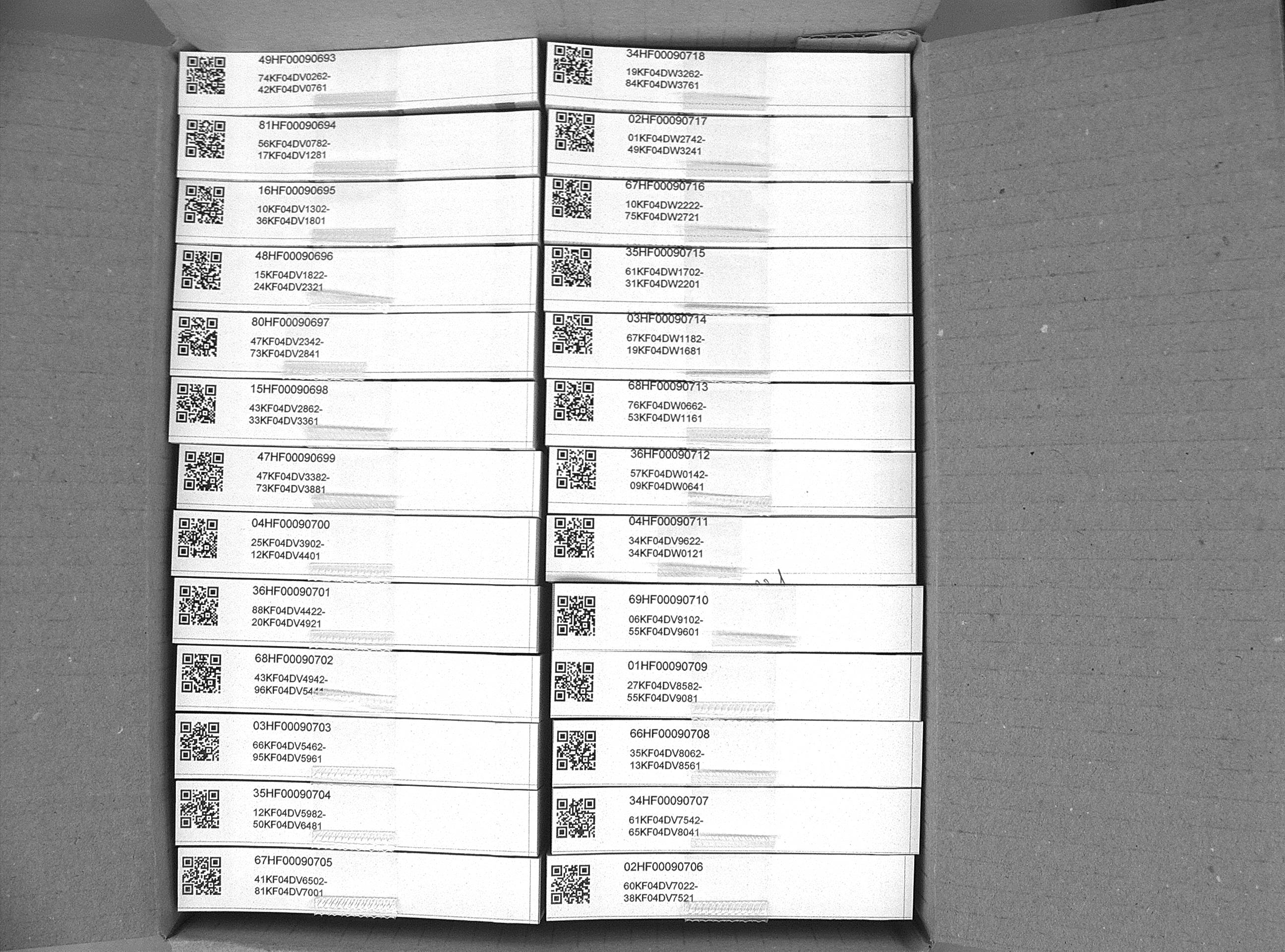

Supplement: Supplementary file 1 [file jimaging-06-00067-s001.zip › QR4f.jpg]
